# Lack of association between *miR-146a* rs2910164 C/G locus and colorectal cancer: from a case–control study to a meta-analysis

**DOI:** 10.1042/BSR20191729

**Published:** 2021-01-04

**Authors:** Jiakai Jiang, Sheng Zhang, Weifeng Tang, Zhiyuan Qiu

**Affiliations:** 1 Department of General Surgery, Changzhou No. 3 People’s Hospital, Changzhou, Jiangsu Province, China; 2 Department of Cardiothoracic Surgery, Affiliated People’s Hospital of Jiangsu University, Zhenjiang, Jiangsu Province, China; 3 Department of Medical Oncology, Affiliated People’s Hospital of Jiangsu University, Zhenjiang, Jiangsu Province, China

**Keywords:** Colorectal cancer, Meta-analysis, MiR-146a, Polymorphism, Risk

## Abstract

Previous studies suggested that *miR-146a* rs2910164 (C/G) locus was predicted to influence the risk of cancer. However, the relationship of *miR-146a* rs2910164 locus with colorectal cancer (CRC) susceptibility was controversial. We recruited 1003 CRC patients and 1303 controls, and performed a case–control study to clarify the correlation of *miR-146a* rs2910164 locus with CRC risk. Subsequently, a comprehensive meta-analysis was conducted to verify our findings. In the case–control study, we suggested that *miR-146a* rs2910164 variants did not alter CRC risk (CG vs. CC: adjusted *P*=0.465; GG vs. CC: adjusted *P*=0.436, CG/GG vs. CC: adjusted *P*=0.387 and GG vs. CC/CG: adjusted *P*=0.589), even in subgroup analysis. Next, we conducted a pooled-analysis to identify the correlation of *miR-146a* rs2910164 locus with CRC risk. In this pooled-analysis, 7947 CRC cases and 12,168 controls were included. We found that *miR-146a* rs2910164 polymorphism did not influence the risk of CRC (G vs. C: *P*=0.537; GG vs. CC: *P*=0.517, CG/GG vs. CC: *P*=0.520 and GG vs. CC/CG: *P*=0.167). Our findings suggest that *miR-146a* rs2910164 C/G polymorphism is not correlated with the susceptibility of CRC. In the future, more case–control studies are needed to confirm our results.

## Introduction

In 2018, it was reported that over 1.8 million new colorectal cancer (CRC) cases were diagnosed and 881,000 CRC-related deaths occurred worldwide [1]. In China, CRC ranked both fifth in terms of malignancy incidence and mortality [2]. Trend of cancer incidence indicated that a significant upward trend was found for CRC in developing countries [3]. Previous studies attributed CRC to aging, unhealthy lifestyle and environmental factors [4–7]. It should be very important to understand susceptibility factors of CRC as they could be beneficial for the evaluation of prevention strategies and susceptibility. Accumulating evidences have shown that an individual’s inherited factors also contribute to the development of CRC.

*MicroRNA* (*miR-*) involves ∼22 nucleotides. Recently, *miRs* have been widely investigated and accepted to be implicated in a number of human diseases, especially in malignancy. Adami *et al.* indicated that *miR-146a* was overexpressed in primary gastric tumor tissues without the status of chronic *Helicobacter pylori* infection and expressed a related lower level in progressed gastric cancer [8]. In addition, a recent study reported that *miR-146a* expression level decreased in progressed CRC patients with lymph node metastasis [9]. Thus, *miR-146a* was considered to be implicated in the development and progression of cancer [10,11].

In the past decade, several studies have focused on the correlation of *miR-146a* rs2910164 C/G with various malignancy susceptibility. *MiR-146a* rs2910164 C/G locus was suggested to be relevant to an increase susceptibility of CRC in several studies [12,13]. However, two publications suggested that *miR-146a* rs2910164 G allele had a lower susceptibility of CRC compared to those with C allele [14,15]. Notably, several pooled-analyses were performed regarding the relationship of CRC risk with this polymorphism [16–23]. However, the previous observations were diversity or even conflicting. Rong *et al.* and Liu *et al.* conducted meta-analyses and found that *miR-146a* rs2910164 polymorphism may confer risk to CRC [17,19]. Other meta-analyses reported that *miR-146a* rs2910164 was not related to CRC [16,18,20–23]. The controversial findings might be due to the limited sample sizes. The aim of the present study was to explore the potential correlation of *miR-146a* rs2910164 polymorphism with CRC risk more extensively. We first conducted a case–control study focusing on the correlation of *miR-146a* rs2910164 with the risk to CRC. Subsequently, we carried out a meta-analysis to further determine the relationship between *miR-146a* rs2910164 locus and CRC risk.

## Materials and methods

### Case–control study

We recruited 1003 sporadic CRC patients between October 2014 and August 2017. They were diagnosed via pathology in Fujian and Jiangsu provinces (China). The mean age of CRC patients was 61.10 ± 12.17 years. In addition, 1303 healthy subjects with a mean age of 61.40 ± 9.61 years were included as controls. The CRC cases and controls were matched by sex and age. The detailed information have been presented in our previous study [24–27]. After giving a written consent in the present study, all participants were questioned the information of risk factor and individual history. The Ethics Committee of Jiangsu University approved protocol of the present study.

Ethylenediamine tetraacetic acid-anticoagulated blood sample was stored in a −80°C refrigerator. Genomic DNA was isolated from lymphocytes by using a DNA Kit (Promega, Madison, U.S.A.). *MiR-146a* rs2910164 variants were analyzed by a SNPscan Kit. To confirm the obtained results, 4% DNA samples were randomly selected and reciprocally tested by another person. The reproducibility of genotyping was 100%.

Genotype frequency of *miR-146a* rs2910164 variants and risk factors were compared by using *χ^2^* test. Crude and adjusted odds ratios (ORs) and 95% confidence intervals (95% CIs) were calculated to predict the association of the *miR-146a* rs2910164 variants with risk to CRC. A *P*<0.05 (two-tailed) was used to determine the significance. Hardy–Weinberg equilibrium (HWE) was used to confirm whether the distribution of *miR-146a* rs2910164 variants accorded with Mendelian inheritance patterns by a Chi-square software. All the comparisons were conducted using SAS 9.4 version software (SAS Institute, Cary, U.S.A.).

### Meta-analysis

To verify the correlation between *miR-146a* rs2910164 locus and the risk of CRC, we subsequently carried out a systematic review and meta-analysis. All studies focusing on the association of *miR-146a* rs2910164 polymorphism with CRC were identified by a widely internet-based search of China Biology Medicine (CBM), PubMed and Embase databases (published up to March 31, 2019) with the terms of: (miR-146a or hsa-mir-146a or miRNA-146a or MicroRNA-146a or rs2910164) and (colorectal or colon or rectum) and (polymorphism or SNP or variant) and (cancer or carcinoma). Other potential papers were obtained by searching of the references of in eligible publications.

The following included criteria were used: (a) studies based on the association between *miR-146a* rs2910164 and CRC risk; (b) genotype data were presented; and (c) the genotype distribution in controls consistent with HWE. Two authors (S. Zhang and J. Jiang) extracted the following information independently: the surname of first author, year of publication, country, ethnicity of participant, the region of CRC, method of genotyping and frequencies of *miR-146a* rs2910164 variants in cases and controls.

Crude ORs with their 95% CIs were harnessed to evaluate the strength of correlation between *miR-146a* rs2910164 and CRC risk. Chi-square-based Cochran’s *Q*-test and *I^2^* test were carried out to assess the heterogeneity among the eligible studies, which were defined as statistical significance if *I^2^*>50% or *P*<0.1. The pooled ORs were calculated by a random-effect model (DerSimonian and Laird method), if we found significant heterogeneity [28,29]. Otherwise, the fixed effects model (the Mantel–Haenszel method) was used [30]. Sensitivity analysis was conducted by one-way method, which removed an individual study in turn to address the stability of our findings under all genetic models. In addition, Begg’s test and Egger’s test were used to assess the bias of publication. A *P*<0.1 was defined as the level for significance of publication bias. The Newcastle–Ottawa Scale (NOS) was used to assess the quality of the included studies. Galbraith radial plot was harnessed to identify the source of heterogeneity. All statistical tests were conducted by using the STATA software (Version 12.0; Stata Corporation, College Station, Texas). A *P* values (two-sided) less than 0.05 was defined as statistically significant association between *miR-146a* rs2910164 and CRC risk. By using Power and Sample Size Calculator software [31], power value (*α* = 0.05) of this meta-analysis was calculated.

## Results

### Case–control study

This case–control study consisted of participants enrolled in a study conducted in Jiangsu and Fujian provinces (China) from October 2014 to August 2017. The detailed information of the present study was described in our previous studies [24,25]. Briefly, patients with 431 colon cancer and 572 rectum cancer were recruited. CRC cases were unrelated and confirmed histologically. Non-cancer subjects were included as controls from the same populations with age and sex matched. And all subjects were unrelated Chinese Han populations. Table 1 lists demographic information and risk factors that were collected in the present study. Table 2 summarizes the corresponding information of *miR-146a* rs2910164. The distribution of *miR-146a* rs2910164 variants in control group is consistent with HWE (*P*=0.706). Data of genotypes and environmental factors are summarized in Supplementary Table S1.

**Table 1:** Distribution of selected characteristics in CRC cases and controls

Variable	Cases (*n*=1003)		Controls (*n*=1303)		*P*
	*n*	%	*n*	%	
Age (years ± standard deviation)	61.10 ± 12.17		61.40 ± 9.61		0.496^*^
Age (years)					0.605^†^
<61	451	44.97	600	46.05	
≥61	552	55.03	703	53.95	
Sex					0.867^†^
Male	620	61.81	801	61.47	
Female	383	38.19	502	38.53	
Smoking	259	25.82	265	20.34	**0.002**^†^
Alcohol use	174	17.35	136	10.44	**<0.001**^†^
BMI (kg/m^2^)					
<24	670	66.80	688	52.80	**<0.001**^†^
≥24	333	33.20	615	47.20	
Site of tumor					
Colon cancer	431	42.97			
Rectum cancer	572	57.03			

* Two-sided student t test;

† Two-sided *χ*^2^ test

Bold values are statistically significant (*P*<0.05).

BMI, body mass index

**Table 2:** Primary information for MiR-146a rs2910164 C/G polymorphism

Genotyped SNPs	MiR-146a rs2910164 C/G
Chromosome	5
Function	non_coding_transcript_variant
Chr Pos (NCBI Build 38)	160485411
MAF^*^ for Chinese in database^†^	0.430
MAF in our controls	0.372
*P* value for HWE^‡^ test in our controls	0.706
Genotyping method	SNPscan
% Genotyping value	98.87%

* MAF: minor allele frequency

† http://gvs.gs.washington.edu/GVS147/

‡ HWE, Hardy–Weinberg equilibrium

Table 3 shows the genotype distribution of *miR-146a* rs2910164 in two groups. *MiR-146a* rs2910164 variant frequencies were 37.55% (CC), 47.96% (CG) and 14.49% (GG) in CRC patients and 39.69% (CC), 46.23% (CG) and 14.08% (GG) in controls. In the studied populations, minor allele frequency (MAF) of *miR-146a* rs2910164 C/G polymorphism was 0.372, which was similar to SNP database for Chinese populations (MAF = 0.430, Table 2). We found that there were no significant association between *miR-146a* rs2910164 polymorphism and susceptibility of CRC (CG vs. CC: crude *P*=0.317; GG vs. CC: crude *P*=0.520, CG/GG vs. CC: crude *P*=0.300; GG vs. CC/CG: crude *P*=0.780). Adjustments for selected risk factors (e.g. age, sex, BMI, smoking and drinking), the null association was also found (CG vs. CC: adjusted *P*=0.465; GG vs. CC: adjusted *P*=0.436, CG/GG vs. CC: adjusted *P*=0.387 and GG vs. CC/CG: adjusted *P*=0.589; Table 3).

**Table 3:** Logistic regression analyses of associations between MiR-146a rs2910164 C/G A polymorphism and risk of CRC

Genotype	Cases (*n*=1003)		Controls (*n*=1303)		Crude OR (95%CI)	*P*	Adjusted OR^*^ (95%CI)	*P*
	*n*	%	*n*	%				
MiR-146a rs2910164								
CC	368	37.55	516	39.69	1.00		1.00	
CG	470	47.96	601	46.23	1.10 (0.92–1.31)	0.317	1.07 (0.89–1.29)	0.465
GG	142	14.49	183	14.08	1.09 (0.84–1.41)	0.520	1.11 (0.86–1.44)	0.436
CG+GG	612	62.45	784	60.31	1.09 (0.92–1.30)	0.300	1.08 (0.91–1.28)	0.387
CC+CG	838	85.51	1,117	85.92	1.00		1.00	
GG	142	14.49	183	14.08	1.03 (0.82–1.31)	0.780	1.07 (0.84–1.36)	0.589
G allele	754	38.47	967	37.19				

* Adjusted for age, sex, BMI, alcohol use and smoking status.

In subgroup analysis, we found that *miR-146a* rs2910164 variants did not alter the susceptibility of colon cancer (CG vs. CC: adjusted *P*=0.863; GG vs. CC: adjusted *P*=0.880, CG/GG vs. CC: adjusted *P*=0.846 and GG vs. CC/CG: adjusted *P*=0.926; Table 4) or rectal cancer (CG vs. CC: adjusted *P*=0.420; GG vs. CC: adjusted *P*=0.337, CG/GG vs. CC: adjusted *P*=0.324 and GG vs. CC/CG: adjusted *P*=0.484; Table 4).

**Table 4:** Stratified analyses between MiR-146a rs2910164 polymorphism and CRC risk by site of tumor

Genotype	Controls (*n*=1303)		Colon cancer cases (*n*=431)		Crude OR (95%CI)	*P*	Adjusted OR^*^ (95%CI)	P^*^	Rectum cancer cases (*n*=572)		Crude OR (95%CI)	*P*	Adjusted OR^*^ (95%CI)	P^*^
	*n*	%	*n*	%					n	%				
MiR-146a rs2910164														
CC	516	39.69	165	39.01	1.00		1.00		203	36.45	1.00		1.00	
CG	601	46.23	200	47.28	1.04 (0.82–1.32)	0.742	1.02 (0.80–1.30)	0.863	270	48.47	1.14 (0.92–1.42)	0.230	1.10 (0.88–1.37)	0.420
GG	183	14.08	58	13.71	0.99 (0.70–1.40)	0.960	1.03 (0.73–1.45)	0.880	84	15.08	1.17 (0.86–1.58)	0.322	1.17 (0.85–1.59)	0.337
CG+GG	784	60.31	258	60.99	1.03 (0.82–1.29)	0.803	1.02 (0.82–1.28)	0.846	354	63.55	1.15 (0.94–1.41)	0.188	1.11 (0.90–1.37)	0.324
CC+CG	1117	85.92	365	86.29	1.00		1.00		473	84.92	1.00		1.00	
GG	183	14.08	58	13.71	0.97 (0.71–1.33)	0.852	1.02 (0.74–1.40)	0.926	84	15.08	1.08 (0.82–1.43)	0.572	1.11 (0.83–1.47)	0.484
G allele	967	37.19	316	37.35					438	39.32				

* Adjusted for age, sex, smoking status, alcohol use and BMI status.

### Meta-analysis of MiR-146a rs2910164 locus and CRC

**Figure 1:**
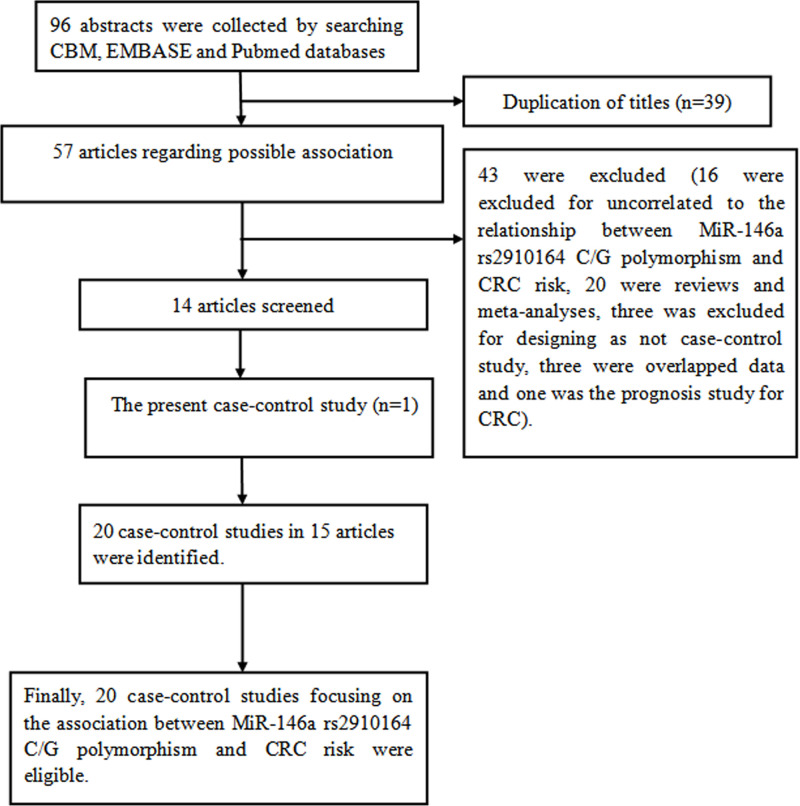
Flow diagram of the meta-analysis of the association between *MiR-146a* rs2910164 polymorphism and CRC risk

Next, we conducted a pooled-analysis to identify the correlation of *miR-146a* rs2910164 with CRC risk. At first, 96 abstracts were collected by searching CBM, EMBASE and Pubmed databases. Figure 1 summarizes the process of meta-analysis. Some publications contained stratified analysis, we treated these subgroups separately [14,32–34]. Table 5 shows the characteristics and *miR-146a* rs2910164 genotypes of included case-control studies. Finally, 7947 CRC cases and 12,168 controls were included to analyze the relationship of *miR-146a* rs2910164 with CRC risk [12–15,32–41].

**Table 5:** Characteristics and genotypes of the included studies in meta-analysis for MiR-146a rs2910164 polymorphism

Study	Publication year	Ethnicity	Country	Region of CRC	Sample size (case/control)	Genotype method	Case			Control			HWE	NOS
							GG	CG	CC	GG	CG	CC		
Hezova et al.	2012	Caucasians	Czech Republic	Mixed	197/122	Taqman	115	70	12	124	79	9	Yes	7
Min et al.	2012	Asians	Korea	Colon	251/502	PCR-RFLP	31	144	76	69	245	188	Yes	7
Min et al.	2012	Asians	Korea	Rectum	184/502	PCR-RFLP	28	87	69	69	245	188	Yes	7
Chae et al.	2013	Asians	Korea	Rectum	176/568	PCR-RFLP	23	87	66	121	282	165	Yes	7
Chae et al.	2013	Asians	Korea	Colon	221/568	PCR-RFLP	38	93	90	121	282	165	Yes	7
Lv et al.	2013	Asians	China	Mixed	353/540	PCR-RFLP	54	230	47	96	274	143	Yes	7
Ma et al.	2013	Asians	China	Rectum	588/1,203	Taqman	229	CG/CC : 359^*^	–	397	CG/CC 806^*^	–	Yes	7
Ma et al.	2013	Asians	China	Colon	559/1,203	Taqman	215	CG/CC : 344^*^	–	397	CG/CC 806^*^	–	Yes	7
Vinci et al.	2013	Caucasians	Italy	Mixed	160/160	HRM	86	57	17	100	65	13	Yes	5
Kupcinskas et al.	2014	Caucasians	Lithuania and Latvia	Mixed	193/428	Taqman	140	50	2	275	134	15	Yes	7
Mao et al.	2014	Asians	China	Mixed	554/566	SNPscan	70	291	186	85	271	205	Yes	9
Hu et al.	2014	Asians	China	Mixed	276/373	PCR-RFLP	34	82	84	44	187	142	Yes	7
Dikaiakos et al.	2015	Caucasians	Greece	Mixed	157/299	PCR-RFLP	8	48	101	21	120	158	Yes	7
Ying et al.	2016	Asians	China	Mixed	1358/1079	MassARRAY	223	655	473	163	529	383	Yes	6
Zhang et al.	2016	Asians	China	Mixed	106/53	PCR-RFLP	28	62	16	10	25	18	Yes	6
Lindor et al.	2017	Caucasians	Australia, U.S.A. and Canada	Mixed	899/204	Not available	G: 1390		C: 408	G: 320		C: 88	Yes	5
Chayeb et al.	2018	Caucasians	Tunisia	Rectum	57/161	PCR-RFLP	20	31	6	49	89	23	Yes	8
Chayeb et al.	2018	Caucasians	Tunisia	Colon	95/161	PCR-RFLP	40	46	9	49	89	23	Yes	8
Gao et al.	2018	Asians	China	Mixed	560/780	PCR-RFLP	130	285	145	149	411	220	Yes	7
Our study	2019	Asians	China	Colon	431/1303	SNPscan	58	200	165	183	601	516	Yes	6
Our study	2019	Asians	China	Rectum	572/1303	SNPscan	84	270	203	183	601	516	Yes	6

* indicates CG/CC

HWE, Hardy–Weinberg equilibrium

**Figure 2:**
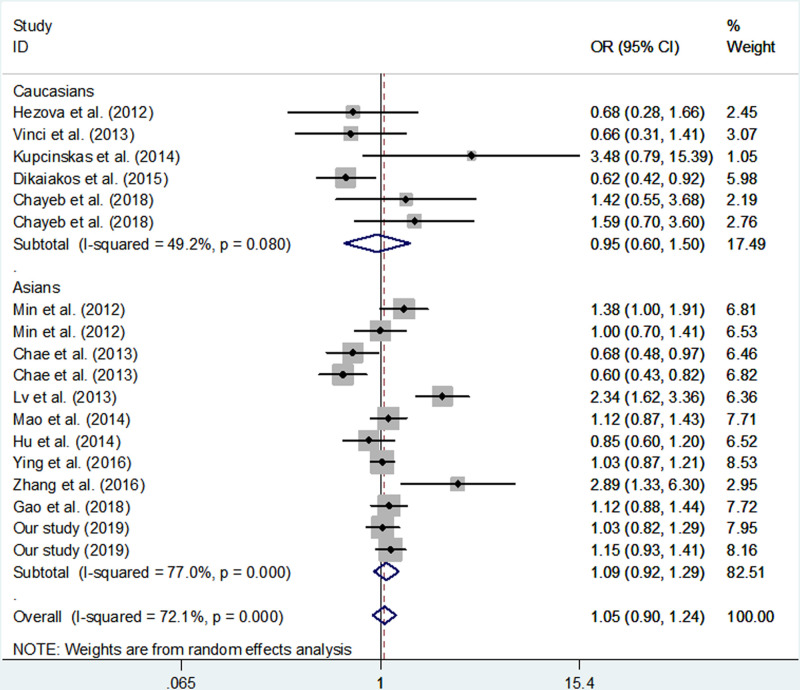
Meta-analysis of the association between *MiR-146a* rs2910164 polymorphism and CRC risk (GG+CG vs. CC, random-effects model)

We found that there was no significant association between *miR-146a* rs2910164 polymorphism and the risk of CRC (G vs. C: *P*=0.537; GG vs. CC: *P*=0.517, CG/GG vs. CC: *P*=0.520 and GG vs. CC/CG: *P*=0.167; Table 6 and Figure 2). Additionally, subgroup analyses were performed by the ethnicity and region of CRC. Null association between *miR-146a* rs2910164 and the susceptibility of CRC was also found (Table 6).

**Table 6:** Meta-analysis of the MiR-146a rs2910164 polymorphism and CRC risk

	No. of study	G vs. C			GG vs. CC			GG+CG vs. CC			GG vs. CC+CG		
		OR (95%CI)	*P*	*P* (*Q*-test)	OR (95%CI)	*P*	*P* (*Q*-test)	OR (95%CI)	*P*	*P* (*Q*-test)	OR (95%CI)	*P*	*P* (*Q*-test)
Overall	21	1.03 (0.94–1.12)	0.537	<0.001	1.06 (0.89–1.27)	0.517	0.002	1.05 (0.90–1.24)	0.520	<0.001	1.08 (0.97–1.20)	0.167	0.033
Type													
Colon cancer	5	1.01 (0.80–1.28)	0.908	0.006	0.98 (0.65–1.48)	0.914	0.040	1.02 (0.69–1.51)	0.926	0.002	1.07 (0.84–1.35)	0.600	0.069
Rectal cancer	5	0.98 (0.78–1.22)	0.823	0.021	0.95 (0.59–1.51)	0.815	0.024	0.97 (0.74–1.28)	0.853	0.083	1.05 (0.80–1.36)	0.741	0.037
Mixed type of CRC	11	1.06 (0.95–1.17)	0.294	0.021	1.17 (0.92–1.48)	0.192	0.044	1.12 (0.87–1.44)	0.373	<0.001	1.09 (0.97–1.22)	0.132	0.176
Ethnicity													
Asians	14	1.03 (0.94–1.13)	0.547	0.002	1.06 (0.88–1.29)	0.530	0.003	1.09 (0.92–1.29)	0.343	<0.001	1.06 (0.93–1.19)	0.393	0.023
Caucasians	7	1.03 (0.84–1.26)	0.796	0.020	1.09 (0.63–1.87)	0.760	0.083	0.95 (0.60–1.50)	0.835	0.080	1.16 (0.96–1.41)	0.129	0.268
NOS scores													
≥6	19	1.04 (0.95–1.14)	0.396	<0.001	1.08 (0.90–1.30)	0.405	0.002	1.07 (0.91–1.26)	0.419	<0.001	1.09 (0.97–1.21)	0.142	0.030
< 6	2	0.91 (0.74–1.12)	0.368	0.712	0.66 (0.30–1.43)	0.291	–	0.66 (0.31–1.41)	0.286	–	0.91 (0.59–1.39)	0.654	–

**Figure 3:**
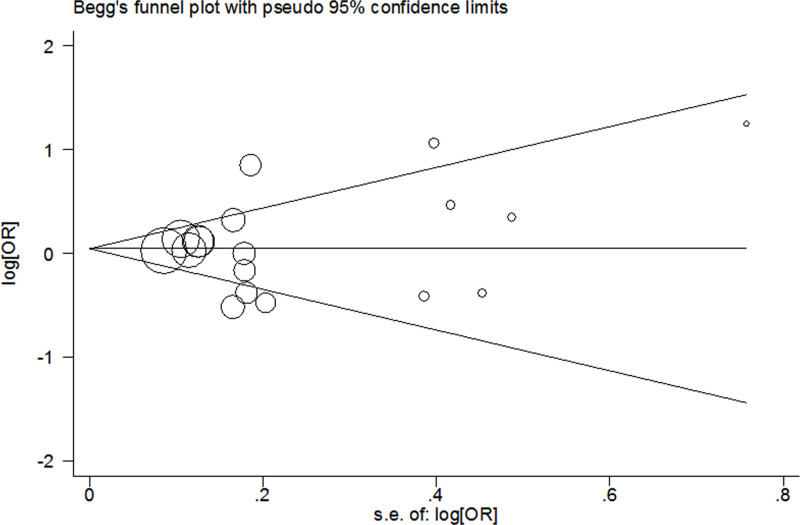
Begg’s funnel plot of meta-analysis (GG+CG vs. CC, random-effects model)

CRC, colorectal cancer;

In this analysis, publication bias was assessed by using Begg’s test and Egger’s linear regression test. And no significant bias was found in any genetic model (Begg’s test: G vs. C: *P*=0.820; GG vs. CC: *P*=0.649; GG/CG vs. CC: *P*=0.649; GG vs. CC/CG: *P*=0.381; Egger’s linear regression test: G vs. C: *P*=0.980; GG vs. CC: *P*=0.761; GG/CG vs. CC: *P*=0.686; GG vs. CC/CG: *P*=0.215; Figure 3).

**Figure 4:**
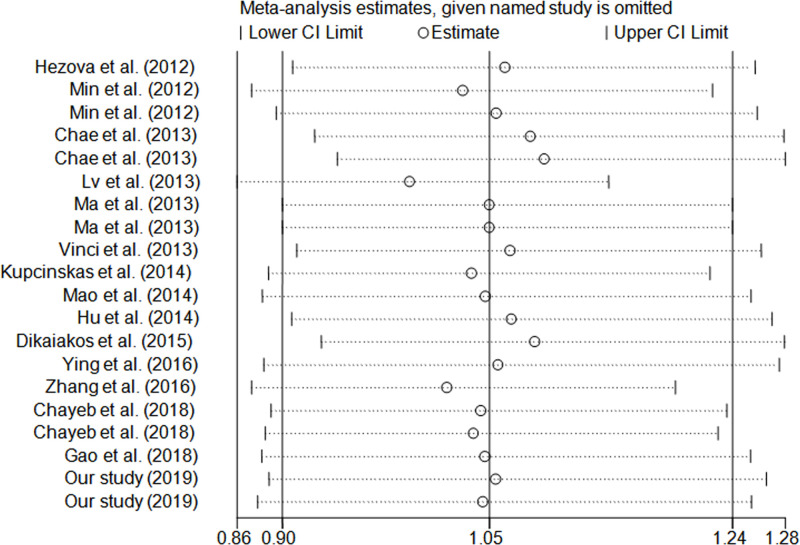
Sensitivity analysis in dominant model (random-effects estimates)

We used one-way method to evaluate the sensitivity of this analysis. The results suggested that any individual study could not materially alter the association between *miR-146a* rs2910164 and CRC risk (Figure 4).

**Figure 5:**
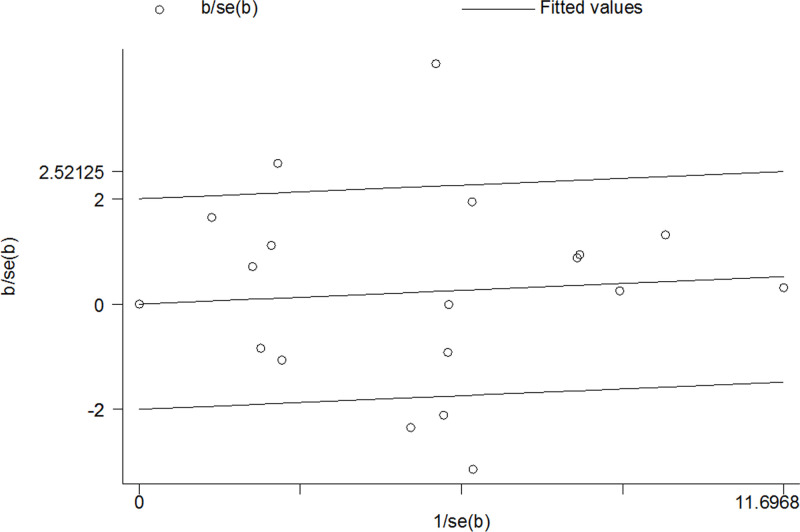
Galbraith radial plot of meta-analysis (GG+CG vs. CC, random-effects model)

Since significant heterogeneity among the studies was found, we conducted subgroup analyses and Galbraith radial plot to identify the source of heterogeneity in a dominant model. Because ethnicity and type of CRC could affect the observations of meta-analysis, subgroup analyses were carried out (Table 6). We found that rectal cancer and Asians might lead to major source of heterogeneity. In Galbraith radial plot, we could find five outliers [12,13,35–37], which contributed significant heterogeneity to this analysis (Figure 5).

NOS scores of the included studies were summarized in Table 5. According to the criterion mentioned in previous study [42,43], we found that only two investigations were poor quality studies [36,44]. When we omitted these poor quality investigations, the findings were not changed.

For this pooled-analysis, the power value (*α* = 0.05) was 0.258 in G vs. C, 0.235 in GG vs. CC, 0.275 in GG/CG vs. CC and 0.579 in GG vs. CC/CG genetic model.

## Discussion

In CRC patients, *miR-146a* expression level decreased in progressed tumors [9]. Since *miR-146a* plays important roles in the development of CRC, we presumed that *miR-146a* rs2910164 variants could influence the susceptibility to CRC. Here, we first performed a case–control study and subsequently conducted a meta-analysis on the potential correlation between *miR-146a* rs2910164 polymorphism and the susceptibility of CRC. In the case–control study, we recruited 1003 sporadic CRC patients and 1303 controls. In meta-analysis, the present study included 21 independent studies with 20,115 subjects (7947 CRC patients and 12,168 controls). Nevertheless, null correlation has been identified between *miR-146a* rs2910164 locus and the risk of CRC, even in different subgroup.

To date, some studies have been carried out to assess this potential correlation between *miR-146a* rs2910164 polymorphism and CRC susceptibility in different populations. However, conflicting findings were found. Consequently, we designed a case–control study matching with age and sex. We got a null correlation between this SNP and the risk of CRC. These findings were similar to some previous publications, which the genotype frequency of the *miR-146a* rs2910164 G allele had no significant difference in CRC cases versus non-cancer controls [35,36,39,40]. It was worth mentioning that the paper was published by Ying *et al.* [40], where the investigation included more than 1000 CRC patients and 1000 controls. However, other publications, identified a significant associations or even suggested opposite findings. Dikaiakos *et al.* [15] and Chae *et al.* [14] reported a decreased risk of the *miR-146a* rs2910164 G allele to CRC. Recently, two publications found an association of the increased risk with *miR-146a* rs2910164 G allele [12,13]. It could be interpreted that these investigations were carried out in different ethnicity, region, age and with variable sample size. A recent meta-analysis has found an association for the European populations in the recessive model [45]. Compared with the meta-analysis mentioned above, the current meta-analysis has included more case–control studies with larger sample sizes. Thus, our findings might be more credible.

In the large-scale pooled-analysis, we found that *miR-146a* rs2910164 might not confer the risk to CRC. However, the interaction between genes and environment factors might be implicated in the occurrence of CRC. Reviewing early publications, some reasons were identified for the conflicting findings. First, different ethnicity may lead to the different results, since the genotype frequencies of the *miR-146a* rs2910164 were diverse between Asians and Caucasians. For example, in the present study, MAF value of *miR-146a* rs2910164 (C/G) was 0.404 in Asians, while the value was 0.631 in Caucasians. On the other hand, most of the included case–control studies did not focus on some vital environmental risk factors (e.g., low intake of dietary fiber, alcohol consumption, tobacco use, obesity, overweight and being physically inactive). It is possible that *miR-146a* rs2910164 has influence the development of CRC, while those environmental factors mentioned above may cover up the role of C→G variation in rs2910164. Thus, in the future, more case–control studies should be designed to explore the relationship of *miR-146a* rs2910164 on the risk of CRC, especially focusing on the interaction of gene–environmental factors.

In the present study, heterogeneity might be noted, which could significantly affect the explaination of our findings. When we carried out subgroup analyses, significant heterogeneity was found in colon cancer and Asians subgroups. Combined the Galbraith radial plot (Figure 5) and the forest plot (Figure 2) in a dominant model, we identified five outliers [12,13,35–37], which might contribute to prominent heterogeneity. The quality evaluation was used to improve the preent study. In the meta-analysis, we found that only two investigations were poor quality study [36,44]. In subgroup analysis, when we omitted them, the findings were not altered materially.

There are, whereas, some potential limitations in the present study. First, this case–control study only focused on Chinese Han populations. Second, for lack of parameter of risk factors, we only calculate the crude ORs and CIs to assess the correlation between *miR-146a* rs2910164 and CRC risk in subsequent meta-analysis. Finally, due to the limited participants in subgroups, the power of subgroup analysis might be insufficient.

To conclude, our findings have not supported a relationship between the *miR-146a* rs2910164 and risk of CRC. The importance of this locus as a genetic predictor for the development of CRC may be very inappreciable and the significance of hereditary screening in healthy individuals may be lack of evidence. In the future, larger studies with well-matched controls are needed to confirm our findings.

## Supplementary Material

Supplementary Table S1

## Data Availability

The genotypes and environmental factors are summarized in Supplementary Table S1.

## Abbreviations

CI: confidence interval
CRC: colorectal cancer
HWE: Hardy–Weinberg equilibrium
OR: odds ratio
