# Supplementary material for: Lack of association between miR-146a rs2910164 C/G locus and colorectal cancer: from a case–control study to a meta-analysis
Source: Biosci Rep. 2021 Jan 4;41(1):BSR20191729. doi: 10.1042/BSR20191729 (PMC7785042; doi:10.1042/BSR20191729)
Supplement: Supplementary Table S1 [file BSR-2019-1729_supp.pdf]

Table S1 Raw data and characteristics of study

Subjects	Sex (male: 1; female: 2)	Age (year)	Smoking(Yes: 1, No: 0)	Drinking (Yes: 1, No: 0)	BMI (≥24: 1, <24: 0)	Region (1: colon cancer, 2:rectal cancer)	rs2910164
CRC-0001	1	71	0	0	1	2	C/C
CRC-0002	1	65	0	0	1	2	C/C
CRC-0003	1	56	0	0	1	1	G/G
CRC-0004	2	57	0	0	0	1	G/C
CRC-0005	2	79	0	0	0	2	G/C
CRC-0006	2	50	0	0	0	2	C/C
CRC-0007	2	38	0	0	0	2	C/C
CRC-0008	1	41	1	1	0	2	G/G
CRC-0009	1	49	1	1	1	2	G/C
CRC-0010	2	51	0	0	0	1	C/C
CRC-0011	2	69	0	0	0	2	C/C
CRC-0012	2	21	0	0	0	1	C/C
CRC-0013	1	46	1	0	0	1	C/C
CRC-0014	1	77	0	0	1	2	C/C
CRC-0015	1	62	0	0	0	2	G/C
CRC-0016	2	70	0	0	0	1	G/C
CRC-0017	2	45	0	0	0	2	G/G
CRC-0018	2	76	0	0	1	2	G/C
CRC-0019	1	33	0	0	0	1	C/C
CRC-0020	1	66	0	0	1	2	C/C
CRC-0021	2	72	0	0	0	1	C/C
CRC-0022	2	55	0	0	0	1	G/C
CRC-0023	1	25	0	0	0	2	C/C
CRC-0024	1	64	1	0	0	1	C/C
CRC-0025	2	56	0	0	1	1	C/C
CRC-0026	1	51	1	1	0	2	C/C
CRC-0027	1	31	0	0	1	1	C/C
CRC-0028	1	38	0	0	0	2	G/C
CRC-0029	1	70	0	0	0	2	C/C
CRC-0030	1	51	0	0	0	2	C/C
CRC-0031	1	59	1	1	1	1	G/G
CRC-0032	2	60	0	0	0	1	G/C
CRC-0033	1	74	1	1	0	1	G/C
CRC-0034	1	59	0	1	0	1	C/C
CRC-0035	1	56	1	1	0	2	G/G
CRC-0036	2	59	0	0	1	2	C/C
CRC-0037	1	39	1	1	0	2	G/C
CRC-0038	2	51	0	0	0	2	G/C
CRC-0039	2	65	0	0	1	2	C/C
CRC-0040	2	67	0	0	0	1	G/C
CRC-0041	2	62	0	0	0	2	G/C
CRC-0042	2	78	0	0	0	2	G/C
CRC-0043	1	54	0	0	0	1	G/G
CRC-0044	2	64	0	0	1	1	G/G
CRC-0045	1	52	0	0	0	2	G/C
CRC-0046	1	62	0	0	1	1	C/C
CRC-0047	2	24	0	0	0	1	C/C
CRC-0048	2	64	0	0	1	1	G/C
CRC-0049	2	77	0	0	1	2	G/G
CRC-0050	2	58	0	0	1	1	C/C
CRC-0051	1	65	1	1	0	1	G/C
CRC-0052	2	83	0	0	0	1	G/C
CRC-0053	1	60	0	0	0	2	C/C
CRC-0054	2	61	0	0	1	2	C/C
CRC-0055	1	44	0	0	0	2	G/C
CRC-0056	1	70	0	0	0	1	C/C
CRC-0057	1	67	0	0	0	2	G/C
CRC-0058	2	35	0	0	0	2	C/C

CRC-0059	1	53	0	1	0	1	G/C
CRC-0060	2	60	0	0	1	2	G/C
CRC-0061	2	37	0	0	0	1	C/C
CRC-0062	1	61	0	0	0	2	G/C
CRC-0063	1	50	1	1	0	1	G/C
CRC-0064	1	79	0	0	0	1	G/C
CRC-0065	2	74	0	0	0	1	G/C
CRC-0066	1	66	0	1	0	1	C/C
CRC-0067	2	54	0	0	0	1	C/C
CRC-0068	1	61	1	1	1	1	C/C
CRC-0069	2	63	0	0	1	1	G/C
CRC-0070	2	30	0	0	0	2	C/C
CRC-0071	1	56	1	0	0	1	G/C
CRC-0072	1	69	0	0	0	2	G/G
CRC-0073	2	62	0	0	0	2	G/C
CRC-0074	2	34	0	0	0	1	G/C
CRC-0075	2	49	0	0	0	1	C/C
CRC-0076	1	74	0	0	1	2	G/C
CRC-0077	2	60	0	0	0	1	C/C
CRC-0078	1	53	1	1	1	2	G/C
CRC-0079	1	41	0	1	1	1	G/C
CRC-0080	2	66	0	0	1	2	G/C
CRC-0081	2	73	0	0	0	2	G/G
CRC-0082	1	70	0	0	1	1	G/C
CRC-0083	2	54	0	0	0	2	G/C
CRC-0084	2	63	0	0	0	1	C/C
CRC-0085	2	63	0	0	0	2	?
CRC-0086	1	70	1	1	0	2	G/C
CRC-0087	2	51	0	0	0	2	G/C
CRC-0088	1	79	0	0	0	2	G/C
CRC-0089	1	61	1	1	0	1	G/C
CRC-0090	2	41	0	0	0	2	G/C
CRC-0091	2	80	1	0	0	2	G/G
CRC-0092	2	77	0	0	0	1	G/C
CRC-0093	1	58	1	0	0	2	C/C
CRC-0094	1	50	1	1	0	2	C/C
CRC-0095	2	53	0	0	0	2	C/C
CRC-0096	2	78	0	0	0	1	C/C
CRC-0097	1	66	1	1	0	1	C/C
CRC-0098	1	51	0	0	1	1	C/C
CRC-0099	1	68	1	1	0	2	G/G
CRC-0100	1	65	0	0	0	1	G/C
CRC-0101	2	53	0	0	1	2	G/C
CRC-0102	1	56	0	0	1	2	G/C
CRC-0103	1	51	1	1	1	2	C/C
CRC-0104	1	69	0	0	0	2	C/C
CRC-0105	1	53	0	1	1	2	G/C
CRC-0106	2	72	0	0	0	1	C/C
CRC-0107	1	51	0	1	0	2	G/C
CRC-0108	1	62	1	1	0	2	C/C
CRC-0109	1	43	1	0	1	2	G/G
CRC-0110	2	65	0	0	0	1	C/C
CRC-0111	1	71	0	0	0	1	G/G
CRC-0112	2	65	0	0	0	2	G/C
CRC-0113	1	74	1	1	0	2	G/C
CRC-0114	1	72	0	0	0	2	G/C
CRC-0115	1	54	0	1	1	2	G/G
CRC-0116	2	64	0	0	1	2	?
CRC-0117	2	52	0	0	0	1	C/C
CRC-0118	1	70	0	0	0	1	G/C
CRC-0119	1	71	0	1	0	2	G/C
CRC-0120	2	64	0	0	0	1	C/C
CRC-0121	1	73	1	1	0	2	C/C
CRC-0122	1	64	1	0	0	1	G/G
CRC-0123	2	66	0	0	1	2	G/C
CRC-0124	2	49	0	0	0	1	G/C

CRC-0125	2	49	0	0	1	2	C/C
CRC-0126	2	53	0	0	0	2	G/C
CRC-0127	2	80	0	0	0	2	C/C
CRC-0128	1	63	1	0	0	2	G/C
CRC-0129	1	77	1	0	1	1	C/C
CRC-0130	2	79	0	0	1	1	G/C
CRC-0131	1	85	1	0	0	1	G/G
CRC-0132	2	74	0	0	0	2	G/G
CRC-0133	2	75	0	0	0	1	G/C
CRC-0134	1	58	1	1	0	2	G/C
CRC-0135	1	60	1	1	0	2	C/C
CRC-0136	1	70	0	0	0	2	G/G
CRC-0137	1	58	1	1	0	1	C/C
CRC-0138	2	80	0	0	1	2	G/C
CRC-0139	1	85	0	0	0	1	C/C
CRC-0140	2	42	0	0	0	2	C/C
CRC-0141	1	64	1	0	1	1	G/G
CRC-0142	1	74	0	0	1	1	G/C
CRC-0143	1	60	1	0	0	1	G/C
CRC-0144	1	57	0	1	0	2	C/C
CRC-0145	1	38	1	0	1	1	G/C
CRC-0146	1	55	1	1	1	2	G/C
CRC-0147	1	53	1	1	1	1	C/C
CRC-0148	1	62	0	0	1	2	G/G
CRC-0149	2	58	0	0	0	2	C/C
CRC-0150	1	79	0	1	0	2	G/G
CRC-0151	2	58	0	0	0	2	G/G
CRC-0152	2	73	0	0	0	2	G/C
CRC-0153	1	52	1	1	0	2	G/C
CRC-0154	1	49	1	0	0	2	G/G
CRC-0155	2	57	0	0	0	2	C/C
CRC-0156	1	67	1	0	0	2	G/C
CRC-0157	2	62	0	0	0	2	G/G
CRC-0158	1	62	1	0	0	2	C/C
CRC-0159	2	53	0	0	1	2	G/C
CRC-0160	1	55	1	0	0	1	C/C
CRC-0161	1	53	0	0	1	2	G/G
CRC-0162	2	61	0	0	0	1	G/C
CRC-0163	1	43	0	1	0	2	G/G
CRC-0164	1	65	1	0	0	2	G/G
CRC-0165	1	36	1	0	0	2	G/C
CRC-0166	2	61	0	0	0	2	C/C
CRC-0167	1	67	0	0	0	1	G/C
CRC-0168	2	62	0	0	1	1	C/C
CRC-0169	2	84	0	0	1	1	C/C
CRC-0170	1	71	1	1	0	1	G/C
CRC-0171	1	69	0	0	0	2	G/C
CRC-0172	1	70	0	0	0	1	G/C
CRC-0173	1	53	0	0	0	1	G/G
CRC-0174	2	56	0	0	0	1	G/C
CRC-0175	2	61	0	0	0	2	G/C
CRC-0176	2	43	0	0	0	2	G/C
CRC-0177	2	57	0	0	1	2	G/G
CRC-0178	1	65	1	0	0	2	G/C
CRC-0179	2	66	0	0	1	1	G/C
CRC-0180	2	56	0	0	0	1	G/C
CRC-0181	1	40	0	1	0	1	C/C
CRC-0182	1	68	1	1	1	2	C/C
CRC-0183	1	53	0	0	0	2	C/C
CRC-0184	1	55	0	1	0	1	G/C
CRC-0185	2	54	0	0	0	2	C/C
CRC-0186	1	53	0	0	0	2	C/C
CRC-0187	1	67	1	1	1	1	G/C
CRC-0188	1	60	0	1	0	2	C/C
CRC-0189	1	58	1	0	1	1	C/C
CRC-0190	1	63	1	1	0	2	C/C

CRC-0191	2	58	0	0	0	1	G/G
CRC-0192	2	57	0	0	0	2	C/C
CRC-0193	2	61	0	0	0	2	G/C
CRC-0194	1	69	0	0	0	2	G/C
CRC-0195	2	56	0	0	0	1	G/C
CRC-0196	1	28	0	1	0	2	G/G
CRC-0197	1	62	0	0	1	1	C/C
CRC-0198	1	73	1	0	0	1	C/C
CRC-0199	1	72	1	1	1	1	G/C
CRC-0200	2	56	0	0	0	1	C/C
CRC-0201	1	44	1	0	1	1	C/C
CRC-0202	1	58	0	1	0	2	G/C
CRC-0203	1	49	0	0	1	2	C/C
CRC-0204	1	40	0	0	0	1	G/C
CRC-0205	2	50	0	0	1	1	G/C
CRC-0206	2	52	0	0	0	2	G/C
CRC-0207	1	57	0	0	1	2	C/C
CRC-0208	2	69	0	0	0	1	C/C
CRC-0209	2	69	0	0	1	2	G/C
CRC-0210	1	67	1	0	1	1	G/C
CRC-0211	1	63	0	1	1	2	C/C
CRC-0212	2	36	0	0	0	2	G/G
CRC-0213	2	50	0	0	1	2	G/C
CRC-0214	1	65	0	0	1	2	C/C
CRC-0215	1	52	0	0	1	2	G/C
CRC-0216	1	65	0	1	1	2	G/C
CRC-0217	2	48	0	0	0	1	G/C
CRC-0218	2	62	0	0	1	1	G/C
CRC-0219	1	67	0	0	1	2	C/C
CRC-0220	1	62	0	1	0	2	G/C
CRC-0221	1	69	1	1	0	2	C/C
CRC-0222	2	64	0	0	0	2	G/C
CRC-0223	1	61	0	0	0	2	G/C
CRC-0224	1	69	1	1	1	2	G/C
CRC-0225	1	46	1	0	1	2	C/C
CRC-0226	1	53	1	0	0	2	G/C
CRC-0227	1	75	1	1	1	2	C/C
CRC-0228	2	65	0	0	0	2	C/C
CRC-0229	1	70	1	0	1	2	G/C
CRC-0230	1	48	1	1	0	2	G/G
CRC-0231	2	74	0	0	0	2	G/G
CRC-0232	2	54	0	0	0	2	G/C
CRC-0233	1	54	0	0	1	2	G/C
CRC-0234	2	76	0	0	0	2	G/C
CRC-0235	2	61	0	0	1	1	G/C
CRC-0236	1	79	0	0	0	2	C/C
CRC-0237	1	67	0	0	0	1	G/C
CRC-0238	1	73	1	0	0	1	G/C
CRC-0239	2	52	0	0	0	2	G/G
CRC-0240	2	72	0	0	0	2	C/C
CRC-0241	1	60	1	0	0	1	G/C
CRC-0242	1	30	0	1	0	2	G/C
CRC-0243	2	43	0	0	0	2	G/C
CRC-0244	2	60	0	0	1	2	G/C
CRC-0245	1	62	0	0	0	1	C/C
CRC-0246	1	46	1	1	0	1	G/C
CRC-0247	2	53	0	0	0	1	C/C
CRC-0248	2	72	0	0	0	1	G/C
CRC-0249	1	58	0	0	0	2	C/C
CRC-0250	2	62	0	0	0	1	G/C
CRC-0251	1	54	1	1	1	2	G/G
CRC-0252	1	66	1	1	0	1	C/C
CRC-0253	2	58	0	0	0	1	C/C
CRC-0254	1	67	0	0	0	1	G/G
CRC-0255	1	46	0	0	0	2	G/C
CRC-0256	1	53	1	1	1	1	G/G

CRC-0257	2	82	0	0	1	1	G/C
CRC-0258	1	73	1	1	0	2	G/C
CRC-0259	2	43	0	0	1	1	G/G
CRC-0260	1	77	0	0	0	2	C/C
CRC-0261	1	61	1	1	0	2	C/C
CRC-0262	2	62	0	0	0	1	G/C
CRC-0263	2	67	0	0	0	1	C/C
CRC-0264	1	52	1	1	0	1	G/C
CRC-0265	2	57	0	0	1	2	G/C
CRC-0266	2	52	0	0	0	1	G/C
CRC-0267	1	51	0	0	0	1	G/C
CRC-0268	1	48	0	0	0	2	G/C
CRC-0269	2	59	0	0	0	2	G/C
CRC-0270	2	58	0	0	0	1	G/G
CRC-0271	1	78	0	0	0	2	G/C
CRC-0272	1	80	1	1	0	2	G/C
CRC-0273	1	47	1	0	0	2	G/G
CRC-0274	1	44	0	0	1	2	G/C
CRC-0275	1	71	0	1	0	1	G/C
CRC-0276	1	41	1	1	0	1	G/C
CRC-0277	1	59	0	1	1	2	C/C
CRC-0278	1	60	1	0	0	1	G/G
CRC-0279	1	64	0	1	1	2	G/G
CRC-0280	2	47	0	0	1	2	G/C
CRC-0281	1	39	0	1	0	2	G/G
CRC-0282	1	60	1	0	0	2	C/C
CRC-0283	1	54	0	1	0	2	C/C
CRC-0284	1	73	0	0	1	1	G/C
CRC-0285	1	58	0	0	1	2	C/C
CRC-0286	1	66	0	0	1	1	C/C
CRC-0287	2	58	0	0	0	2	G/C
CRC-0288	1	50	0	0	0	2	G/C
CRC-0289	1	63	0	0	0	2	G/C
CRC-0290	1	72	1	1	1	2	G/C
CRC-0291	1	67	0	0	1	1	G/C
CRC-0292	1	60	0	0	0	2	C/C
CRC-0293	1	76	0	0	0	2	C/C
CRC-0294	2	61	0	0	1	2	G/C
CRC-0295	2	45	0	0	1	1	G/C
CRC-0296	1	78	0	0	0	2	G/G
CRC-0297	1	66	1	1	0	1	C/C
CRC-0298	2	60	0	0	0	2	G/C
CRC-0299	1	57	0	0	0	2	G/C
CRC-0300	2	64	0	0	0	2	?
CRC-0301	2	49	0	0	0	1	G/C
CRC-0302	1	47	0	0	0	1	G/G
CRC-0303	1	78	0	0	0	2	G/C
CRC-0304	2	51	0	0	0	1	G/C
CRC-0305	2	60	0	0	1	2	G/G
CRC-0306	1	67	1	0	0	2	C/C
CRC-0307	1	62	0	1	0	2	G/C
CRC-0308	1	41	1	1	1	2	C/C
CRC-0309	2	51	0	0	0	1	G/C
CRC-0310	1	47	1	1	0	2	G/C
CRC-0311	2	60	0	0	1	2	G/C
CRC-0312	1	73	1	1	0	1	C/C
CRC-0313	1	68	1	1	1	1	C/C
CRC-0314	1	55	0	0	0	2	G/C
CRC-0315	2	24	0	0	0	2	G/C
CRC-0316	1	59	0	0	0	2	G/C
CRC-0317	2	59	0	0	0	1	G/C
CRC-0318	1	76	0	0	0	1	C/C
CRC-0319	1	45	0	1	1	2	G/C
CRC-0320	2	74	0	0	0	2	G/C
CRC-0321	2	26	0	0	0	2	C/C
CRC-0322	1	60	1	1	1	1	G/C

CRC-0323	1	48	0	0	0	2	G/C
CRC-0324	2	58	0	0	0	2	G/C
CRC-0325	2	71	0	0	1	2	C/C
CRC-0326	1	41	0	0	0	2	C/C
CRC-0327	2	68	0	0	0	2	G/C
CRC-0328	1	68	0	0	0	2	C/C
CRC-0329	1	49	1	1	1	2	G/C
CRC-0330	1	65	1	1	1	1	G/G
CRC-0331	1	47	0	1	1	2	G/C
CRC-0332	1	46	0	0	0	2	G/G
CRC-0333	1	70	1	1	1	2	C/C
CRC-0334	1	79	0	0	1	2	G/C
CRC-0335	1	67	0	0	0	1	C/C
CRC-0336	2	69	0	0	0	1	G/C
CRC-0337	1	49	0	0	0	2	G/C
CRC-0338	1	44	1	1	0	2	G/C
CRC-0339	2	51	0	0	0	2	G/C
CRC-0340	1	50	0	0	1	2	?
CRC-0341	1	35	1	0	0	2	C/C
CRC-0342	2	67	0	0	0	1	C/C
CRC-0343	1	86	1	0	0	2	G/C
CRC-0344	2	61	0	0	0	1	G/C
CRC-0345	2	63	0	0	0	2	C/C
CRC-0346	2	35	0	0	0	1	G/C
CRC-0347	2	42	0	0	0	2	G/C
CRC-0348	1	62	0	1	0	2	G/G
CRC-0349	2	71	0	0	0	2	G/C
CRC-0350	1	73	1	0	0	2	G/C
CRC-0351	1	35	1	0	0	2	G/C
CRC-0352	1	48	0	0	1	2	G/G
CRC-0353	2	74	0	0	0	2	G/C
CRC-0354	1	58	1	1	0	2	C/C
CRC-0355	2	60	0	0	1	2	G/C
CRC-0356	1	66	1	1	0	1	G/C
CRC-0357	1	67	0	1	0	2	G/C
CRC-0358	1	65	0	0	1	2	G/G
CRC-0359	2	69	0	0	0	2	G/C
CRC-0360	2	56	0	0	1	1	G/G
CRC-0361	1	42	0	1	0	2	C/C
CRC-0362	1	47	1	1	1	1	G/C
CRC-0363	2	74	0	0	0	2	G/G
CRC-0364	1	69	1	1	0	1	G/C
CRC-0365	1	62	1	1	0	2	G/C
CRC-0366	1	70	0	0	0	2	G/C
CRC-0367	2	70	0	0	0	2	G/G
CRC-0368	1	48	0	0	0	1	C/C
CRC-0369	1	45	0	0	0	2	G/C
CRC-0370	1	58	0	0	0	1	C/C
CRC-0371	1	77	1	0	0	2	G/G
CRC-0372	1	53	0	0	0	1	G/C
CRC-0373	2	69	0	0	1	1	G/C
CRC-0374	1	82	1	0	0	1	G/C
CRC-0375	2	74	0	0	0	1	C/C
CRC-0376	1	52	0	0	1	2	G/C
CRC-0377	2	66	0	0	0	1	G/C
CRC-0378	1	67	0	0	1	1	G/G
CRC-0379	2	41	0	0	1	1	C/C
CRC-0380	1	60	0	0	0	2	G/C
CRC-0381	1	67	0	0	0	2	G/C
CRC-0382	2	81	0	0	0	2	G/G
CRC-0383	1	51	0	0	0	1	C/C
CRC-0384	1	64	0	0	0	1	C/C
CRC-0385	2	54	0	0	0	2	G/G
CRC-0386	1	60	0	0	0	1	C/C
CRC-0387	1	51	1	1	1	1	G/C
CRC-0388	1	67	0	1	0	2	G/C

CRC-0389	1	84	0	0	0	2	G/C
CRC-0390	1	62	0	0	0	2	G/C
CRC-0391	1	75	1	0	1	2	G/C
CRC-0392	2	74	0	0	1	2	G/C
CRC-0393	1	73	1	0	1	2	G/G
CRC-0394	1	57	0	0	1	1	G/C
CRC-0395	2	63	0	0	0	2	C/C
CRC-0396	2	73	0	0	1	2	G/G
CRC-0397	1	64	0	0	0	2	G/C
CRC-0398	1	64	0	1	0	2	G/C
CRC-0399	1	72	1	0	0	1	G/C
CRC-0400	1	65	1	0	0	2	G/G
CRC-0401	1	45	0	0	1	2	G/C
CRC-0402	2	63	0	0	1	2	C/C
CRC-0403	2	76	0	0	0	1	G/C
CRC-0404	2	83	0	0	0	1	C/C
CRC-0405	2	60	0	0	0	1	C/C
CRC-0406	1	66	0	0	1	2	C/C
CRC-0407	2	70	0	0	0	2	G/C
CRC-0408	1	67	0	0	0	2	G/G
CRC-0409	2	55	0	0	0	1	G/C
CRC-0410	1	60	0	1	1	2	G/C
CRC-0411	1	51	1	0	0	1	G/G
CRC-0412	1	62	0	0	0	2	G/C
CRC-0413	1	77	0	1	1	2	C/C
CRC-0414	1	71	0	0	1	2	C/C
CRC-0415	1	68	0	0	1	2	C/C
CRC-0416	1	69	0	0	1	2	C/C
CRC-0417	2	78	0	0	1	1	G/C
CRC-0418	2	75	0	0	0	2	G/C
CRC-0419	1	60	0	0	1	1	G/C
CRC-0420	1	84	0	0	0	2	C/C
CRC-0421	1	53	0	0	1	1	G/C
CRC-0422	2	44	0	0	0	1	G/C
CRC-0423	1	72	0	0	1	2	C/C
CRC-0424	2	71	0	0	0	1	G/G
CRC-0425	1	68	1	0	0	1	C/C
CRC-0426	1	84	0	0	0	1	C/C
CRC-0427	1	64	0	1	1	1	G/G
CRC-0428	2	56	0	0	1	1	C/C
CRC-0429	2	72	0	0	1	1	G/C
CRC-0430	1	56	1	1	0	2	G/G
CRC-0431	1	84	0	0	1	1	G/C
CRC-0432	2	53	0	0	0	1	G/G
CRC-0433	1	59	0	0	0	1	C/C
CRC-0434	1	58	1	1	1	2	G/C
CRC-0435	1	61	1	0	0	2	G/C
CRC-0436	1	68	0	0	1	2	C/C
CRC-0437	2	47	0	0	1	2	C/C
CRC-0438	1	77	0	0	1	1	C/C
CRC-0439	1	72	0	0	0	2	C/C
CRC-0440	2	60	0	0	0	1	G/C
CRC-0441	1	79	1	1	0	2	C/C
CRC-0442	2	73	0	0	0	1	C/C
CRC-0443	2	68	0	0	0	1	G/C
CRC-0444	2	64	0	0	1	1	G/C
CRC-0445	1	50	1	1	1	1	G/C
CRC-0446	2	68	0	0	1	2	G/C
CRC-0447	2	81	0	0	1	1	C/C
CRC-0448	1	80	0	0	0	1	G/G
CRC-0449	1	60	0	0	0	1	G/G
CRC-0450	1	72	0	0	1	1	G/C
CRC-0451	1	57	0	0	0	1	C/C
CRC-0452	2	75	0	0	1	1	C/C
CRC-0453	1	38	1	0	1	2	G/C
CRC-0454	2	69	0	0	0	1	G/C

CRC-0455	2	76	0	0	0	1	C/C
CRC-0456	1	63	0	0	0	2	G/G
CRC-0457	1	76	1	0	0	1	C/C
CRC-0458	1	81	0	0	0	1	G/C
CRC-0459	1	79	0	0	0	2	C/C
CRC-0460	2	57	0	0	0	1	?
CRC-0461	2	74	0	0	0	1	G/C
CRC-0462	1	77	1	0	1	1	C/C
CRC-0463	1	68	0	0	0	1	G/G
CRC-0464	1	71	0	0	0	2	C/C
CRC-0465	2	78	0	0	1	2	C/C
CRC-0466	1	52	0	0	0	1	G/C
CRC-0467	2	54	0	0	1	1	C/C
CRC-0468	1	54	0	0	1	2	G/G
CRC-0469	1	67	1	0	0	2	C/C
CRC-0470	2	63	0	0	0	1	G/G
CRC-0471	2	70	0	0	1	2	C/C
CRC-0472	2	65	0	0	1	2	G/C
CRC-0473	2	70	0	0	0	2	G/G
CRC-0474	1	56	0	0	0	2	G/C
CRC-0475	1	59	1	0	0	2	C/C
CRC-0476	1	63	0	0	1	2	C/C
CRC-0477	2	62	0	0	0	2	C/C
CRC-0478	1	66	0	0	1	1	G/G
CRC-0479	1	73	0	0	0	1	G/C
CRC-0480	1	69	1	1	0	2	C/C
CRC-0481	2	68	0	0	0	1	C/C
CRC-0482	1	62	0	0	0	1	G/G
CRC-0483	1	60	0	0	0	2	C/C
CRC-0484	1	53	0	0	1	1	C/C
CRC-0485	2	62	0	0	0	2	C/C
CRC-0486	1	64	0	0	1	2	G/C
CRC-0487	2	51	0	0	1	1	G/C
CRC-0488	1	75	1	0	0	2	C/C
CRC-0489	1	62	0	0	1	1	C/C
CRC-0490	1	74	0	0	0	2	C/C
CRC-0491	2	64	0	0	1	1	G/C
CRC-0492	1	73	0	0	1	2	G/G
CRC-0493	1	81	0	0	0	1	G/C
CRC-0494	1	41	0	0	1	2	C/C
CRC-0495	1	67	0	1	1	1	G/C
CRC-0496	2	58	0	0	0	2	G/G
CRC-0497	1	78	0	0	0	1	G/C
CRC-0498	1	45	0	0	1	1	C/C
CRC-0499	2	68	0	0	0	2	G/G
CRC-0500	1	71	0	0	0	1	G/G
CRC-0501	1	60	0	0	0	1	G/C
CRC-0502	2	74	0	0	0	2	G/C
CRC-0503	1	76	1	0	0	2	G/C
CRC-0504	1	75	1	0	0	2	G/C
CRC-0505	1	60	1	1	0	2	?
CRC-0506	2	49	0	0	0	2	C/C
CRC-0507	1	71	1	0	1	2	G/C
CRC-0508	2	65	0	0	0	2	C/C
CRC-0509	1	88	0	0	0	2	?
CRC-0510	1	68	1	0	1	2	G/G
CRC-0511	2	59	0	0	1	1	G/G
CRC-0512	1	51	0	0	0	2	G/C
CRC-0513	1	45	0	0	0	2	C/C
CRC-0514	1	75	1	0	1	2	G/C
CRC-0515	2	54	0	0	1	2	C/C
CRC-0516	1	50	0	0	1	1	G/C
CRC-0517	2	82	0	0	0	2	G/C
CRC-0518	1	43	0	0	0	2	G/C
CRC-0519	1	50	0	0	0	2	?
CRC-0520	1	67	0	0	0	2	G/G

CRC-0521	1	72	0	0	1	1	G/G
CRC-0522	2	62	0	0	1	1	C/C
CRC-0523	1	77	0	0	1	2	G/C
CRC-0524	2	56	0	0	1	2	G/C
CRC-0525	1	75	0	0	0	1	G/G
CRC-0526	1	81	1	1	0	2	G/C
CRC-0527	1	63	0	0	1	1	C/C
CRC-0528	1	73	0	0	1	2	C/C
CRC-0529	2	68	0	0	0	1	C/C
CRC-0530	1	80	1	0	0	1	G/C
CRC-0531	1	46	0	0	1	1	G/C
CRC-0532	1	58	0	0	1	2	G/C
CRC-0533	2	80	0	0	0	2	C/C
CRC-0534	1	37	0	0	1	1	G/C
CRC-0535	1	69	0	0	0	2	C/C
CRC-0536	2	59	0	0	1	1	G/C
CRC-0537	1	69	0	1	0	2	G/G
CRC-0538	1	74	1	1	0	2	G/C
CRC-0539	1	54	0	0	1	1	G/C
CRC-0540	2	53	0	0	1	1	G/G
CRC-0541	1	66	1	1	1	1	G/C
CRC-0542	1	67	0	0	1	1	C/C
CRC-0543	1	50	1	1	0	1	C/C
CRC-0544	2	79	0	0	0	1	G/C
CRC-0545	1	67	0	0	0	2	G/C
CRC-0546	1	66	1	0	0	2	G/C
CRC-0547	1	49	0	0	1	1	C/C
CRC-0548	1	53	0	0	0	2	G/C
CRC-0549	1	68	1	1	0	1	C/C
CRC-0550	1	63	1	0	0	1	C/C
CRC-0551	1	60	0	0	1	2	G/C
CRC-0552	2	78	0	0	0	2	G/C
CRC-0553	2	77	0	0	1	1	G/G
CRC-0554	1	74	0	0	0	1	G/G
CRC-0555	2	77	0	0	1	1	G/C
CRC-0556	1	38	1	0	0	1	G/C
CRC-0557	2	72	0	0	0	1	G/G
CRC-0558	2	46	0	0	0	2	G/G
CRC-0559	1	52	0	0	1	2	C/C
CRC-0560	1	49	0	0	1	2	C/C
CRC-0561	1	62	0	0	1	2	G/C
CRC-0562	1	74	0	0	1	2	G/C
CRC-0563	1	82	0	1	0	1	C/C
CRC-0564	2	72	0	0	0	1	G/C
CRC-0565	2	66	0	0	1	2	G/C
CRC-0566	1	72	0	0	0	2	G/C
CRC-0567	2	64	0	0	0	2	?
CRC-0568	1	79	0	0	0	1	G/C
CRC-0569	1	63	0	0	0	1	G/G
CRC-0570	2	61	0	0	1	2	G/G
CRC-0571	1	67	1	1	0	1	?
CRC-0572	1	67	0	0	1	1	G/C
CRC-0573	1	78	1	0	0	1	G/C
CRC-0574	2	69	0	0	1	2	G/G
CRC-0575	1	79	0	0	0	2	G/C
CRC-0576	1	63	0	0	1	2	G/C
CRC-0577	1	70	0	1	1	2	G/C
CRC-0578	2	44	0	0	0	2	G/C
CRC-0579	1	62	0	0	0	2	?
CRC-0580	1	61	0	1	0	1	C/C
CRC-0581	1	69	0	0	0	1	G/C
CRC-0582	1	63	0	1	0	2	C/C
CRC-0583	1	80	0	0	0	2	G/G
CRC-0584	1	59	1	1	1	1	?
CRC-0585	1	76	0	0	1	2	?
CRC-0586	1	68	0	0	0	2	?

CRC-0587	1	52	0	1	0	1	G/C
CRC-0588	1	74	0	0	0	1	C/C
CRC-0589	1	43	0	0	1	1	G/C
CRC-0590	1	68	0	1	0	2	G/C
CRC-0591	2	87	0	0	0	1	C/C
CRC-0592	2	47	0	0	0	1	C/C
CRC-0593	1	82	1	0	0	1	G/C
CRC-0594	2	67	0	0	0	1	C/C
CRC-0595	2	64	0	0	1	2	G/C
CRC-0596	2	54	0	0	0	1	G/C
CRC-0597	1	62	0	0	1	2	G/G
CRC-0598	1	70	1	1	0	2	C/C
CRC-0599	1	82	0	0	0	1	C/C
CRC-0600	2	61	0	0	0	2	C/C
CRC-0601	2	74	0	0	1	2	G/C
CRC-0602	2	57	0	0	0	2	G/G
CRC-0603	1	60	1	1	0	2	G/C
CRC-0604	2	60	0	0	0	1	C/C
CRC-0605	1	69	0	0	0	1	C/C
CRC-0606	1	62	0	0	0	2	G/C
CRC-0607	1	65	0	0	0	2	C/C
CRC-0608	1	66	0	0	1	2	G/G
CRC-0609	2	47	0	0	0	2	C/C
CRC-0610	1	70	0	0	0	2	G/G
CRC-0611	1	67	1	0	1	2	C/C
CRC-0612	1	63	1	0	0	2	?
CRC-0613	1	53	1	0	1	1	?
CRC-0614	2	66	0	0	0	1	?
CRC-0615	1	63	0	0	1	1	?
CRC-0616	1	41	0	1	1	2	?
CRC-0617	1	90	0	0	0	2	C/C
CRC-0618	2	54	0	0	0	2	C/C
CRC-0619	1	60	1	0	0	1	C/C
CRC-0620	1	53	0	0	1	2	G/C
CRC-0621	2	38	0	0	0	2	G/C
CRC-0622	2	52	0	0	0	2	C/C
CRC-0623	2	74	0	0	1	2	G/C
CRC-0624	2	60	0	0	0	1	C/C
CRC-0625	1	52	0	0	0	2	C/C
CRC-0626	2	47	0	0	0	1	G/C
CRC-0627	2	44	0	0	0	1	G/C
CRC-0628	2	79	0	0	1	1	G/C
CRC-0629	2	73	0	0	1	2	G/C
CRC-0630	2	79	0	0	1	1	C/C
CRC-0631	1	52	1	1	0	2	G/C
CRC-0632	2	68	0	0	0	2	C/C
CRC-0633	2	57	0	0	0	2	G/C
CRC-0634	1	63	1	1	0	2	C/C
CRC-0635	1	45	1	1	0	1	C/C
CRC-0636	2	68	0	0	1	1	C/C
CRC-0637	1	61	1	0	0	1	C/C
CRC-0638	1	59	1	0	1	2	C/C
CRC-0639	1	47	0	0	1	1	C/C
CRC-0640	1	51	0	0	1	1	C/C
CRC-0641	1	68	1	0	0	1	G/C
CRC-0642	2	63	0	0	0	2	C/C
CRC-0643	2	59	0	0	0	2	G/C
CRC-0644	1	59	0	1	0	2	G/C
CRC-0645	1	77	1	0	0	1	G/C
CRC-0646	1	56	1	1	1	2	G/C
CRC-0647	1	79	0	0	1	2	G/C
CRC-0648	1	65	1	0	0	2	G/C
CRC-0649	1	48	1	1	0	2	C/C
CRC-0650	1	76	1	0	0	1	G/C
CRC-0651	1	61	0	0	1	2	C/C
CRC-0652	1	59	1	0	1	2	C/C

CRC-0653	1	68	1	0	1	2	C/C
CRC-0654	1	54	1	0	1	1	G/C
CRC-0655	1	74	1	1	0	2	G/C
CRC-0656	1	58	1	1	0	1	C/C
CRC-0657	1	61	1	0	1	2	G/C
CRC-0658	1	53	1	1	1	2	G/G
CRC-0659	2	65	0	0	0	1	C/C
CRC-0660	2	82	0	0	0	1	G/G
CRC-0661	1	66	1	0	1	2	G/C
CRC-0662	1	59	0	0	0	1	C/C
CRC-0663	2	71	0	0	0	2	G/C
CRC-0664	1	79	1	0	0	1	C/C
CRC-0665	2	51	0	0	0	2	C/C
CRC-0666	2	70	0	0	0	2	G/C
CRC-0667	1	71	0	0	0	1	G/C
CRC-0668	1	79	1	0	0	2	C/C
CRC-0669	2	57	0	0	0	2	C/C
CRC-0670	2	63	0	0	0	2	G/G
CRC-0671	2	53	0	0	0	1	C/C
CRC-0672	1	61	1	1	0	2	G/C
CRC-0673	1	51	0	0	0	2	C/C
CRC-0674	2	51	0	0	0	2	G/G
CRC-0675	1	66	0	0	1	1	C/C
CRC-0676	1	59	0	0	0	2	C/C
CRC-0677	2	75	0	0	0	2	C/C
CRC-0678	1	60	0	0	0	1	G/G
CRC-0679	1	60	1	1	0	2	G/C
CRC-0680	1	63	0	0	1	2	G/C
CRC-0681	1	64	0	0	0	1	G/C
CRC-0682	1	64	1	1	0	1	C/C
CRC-0683	1	47	0	0	1	1	C/C
CRC-0684	2	70	0	0	0	2	G/C
CRC-0685	1	61	0	0	1	1	G/C
CRC-0686	2	84	0	0	0	1	C/C
CRC-0687	1	66	1	0	1	1	?
CRC-0688	2	35	0	0	0	2	G/C
CRC-0689	1	79	1	0	0	1	G/C
CRC-0690	1	58	0	0	1	1	G/C
CRC-0691	1	64	0	0	1	1	G/C
CRC-0692	1	63	1	0	0	1	C/C
CRC-0693	2	67	0	0	1	2	C/C
CRC-0694	2	53	0	0	1	2	G/C
CRC-0695	2	52	0	0	0	2	G/C
CRC-0696	2	43	0	0	0	1	C/C
CRC-0697	2	56	0	0	0	1	C/C
CRC-0698	1	65	1	0	0	2	?
CRC-0699	1	60	1	0	0	2	G/C
CRC-0700	1	69	1	1	0	2	G/C
CRC-0701	1	31	0	0	0	2	G/C
CRC-0702	1	43	0	1	0	1	G/C
CRC-0703	2	64	0	0	0	1	G/C
CRC-0704	1	84	1	1	0	2	C/C
CRC-0705	1	82	0	1	1	1	G/G
CRC-0706	1	70	1	0	1	1	G/C
CRC-0707	2	65	0	0	1	1	C/C
CRC-0708	1	46	1	0	0	2	C/C
CRC-0709	1	55	0	0	0	2	G/C
CRC-0710	1	62	0	0	0	2	G/C
CRC-0711	1	83	0	0	1	2	G/G
CRC-0712	1	66	0	0	1	2	C/C
CRC-0713	1	64	1	0	1	2	G/C
CRC-0714	2	58	0	0	0	2	?
CRC-0715	1	82	1	1	0	2	G/C
CRC-0716	2	78	0	0	0	1	C/C
CRC-0717	1	49	1	1	0	1	C/C
CRC-0718	2	67	0	0	0	2	G/C

CRC-0719	1	59	0	0	1	1	G/C
CRC-0720	2	68	0	0	0	1	G/C
CRC-0721	2	68	0	0	0	1	G/C
CRC-0722	2	36	0	0	0	1	G/G
CRC-0723	2	66	0	0	0	2	G/C
CRC-0724	1	76	1	0	0	2	C/C
CRC-0725	1	70	0	0	0	2	C/C
CRC-0726	1	77	1	0	0	2	C/C
CRC-0727	2	61	0	0	1	2	G/C
CRC-0728	1	30	1	0	0	1	G/C
CRC-0729	1	29	0	0	0	1	G/C
CRC-0730	2	52	0	0	0	2	G/C
CRC-0731	1	62	1	0	1	1	C/C
CRC-0732	2	81	0	0	1	2	G/C
CRC-0733	1	40	0	1	1	2	C/C
CRC-0734	1	66	0	0	1	1	G/G
CRC-0735	1	48	1	0	1	1	G/C
CRC-0736	2	69	0	0	1	1	C/C
CRC-0737	1	77	0	1	0	2	G/C
CRC-0738	2	36	0	0	0	1	G/C
CRC-0739	2	64	0	0	0	2	G/C
CRC-0740	1	72	1	0	0	1	G/G
CRC-0741	2	40	0	0	0	1	C/C
CRC-0742	1	74	1	0	0	2	G/C
CRC-0743	1	63	1	1	1	1	G/G
CRC-0744	2	58	0	0	0	1	C/C
CRC-0745	1	51	0	0	0	1	C/C
CRC-0746	1	70	0	0	0	2	G/C
CRC-0747	1	42	0	0	0	1	G/G
CRC-0748	2	47	0	0	0	2	G/C
CRC-0749	2	81	0	0	0	1	G/C
CRC-0750	1	62	1	0	0	1	G/C
CRC-0751	2	70	0	0	0	2	G/G
CRC-0752	1	73	1	1	0	2	G/C
CRC-0753	2	53	0	0	1	1	G/C
CRC-0754	1	41	0	1	1	2	G/C
CRC-0755	1	57	1	0	1	2	G/C
CRC-0756	1	86	0	0	1	1	G/C
CRC-0757	2	30	0	0	0	1	C/C
CRC-0758	1	73	0	0	0	2	G/G
CRC-0759	1	42	1	0	0	1	C/C
CRC-0760	1	72	0	0	0	2	G/C
CRC-0761	1	70	1	0	0	2	C/C
CRC-0762	2	66	0	0	1	2	G/C
CRC-0763	1	69	0	0	1	1	G/C
CRC-0764	2	71	0	0	1	2	C/C
CRC-0765	1	47	1	0	0	1	G/C
CRC-0766	1	60	1	1	0	2	G/C
CRC-0767	1	86	0	0	0	1	C/C
CRC-0768	2	73	0	0	1	1	C/C
CRC-0769	2	73	0	0	0	1	G/C
CRC-0770	2	58	0	0	0	1	C/C
CRC-0771	1	64	0	1	0	2	G/C
CRC-0772	2	67	0	0	1	2	C/C
CRC-0773	1	69	1	0	1	2	G/G
CRC-0774	1	26	0	0	0	2	G/C
CRC-0775	1	44	1	1	0	2	C/C
CRC-0776	1	66	0	0	1	1	C/C
CRC-0777	1	61	1	1	0	2	G/C
CRC-0778	2	74	0	0	0	1	C/C
CRC-0779	1	67	1	0	0	2	C/C
CRC-0780	1	76	1	0	0	1	G/C
CRC-0781	2	49	0	0	0	1	C/C
CRC-0782	1	51	0	0	1	1	C/C
CRC-0783	2	67	0	0	1	1	C/C
CRC-0784	2	67	0	0	1	2	G/C

CRC-0785	1	39	1	1	0	2	C/C
CRC-0786	2	53	0	0	0	1	C/C
CRC-0787	1	58	0	0	1	1	G/G
CRC-0788	2	63	0	0	0	2	C/C
CRC-0789	2	82	0	0	0	1	G/C
CRC-0790	1	58	1	0	0	1	G/G
CRC-0791	2	56	0	0	1	2	G/C
CRC-0792	1	74	1	1	1	2	C/C
CRC-0793	2	39	0	0	0	2	G/G
CRC-0794	2	58	0	0	1	2	C/C
CRC-0795	1	49	1	1	0	1	C/C
CRC-0796	2	27	0	0	0	1	C/C
CRC-0797	1	80	1	0	0	2	C/C
CRC-0798	1	75	1	0	0	1	G/G
CRC-0799	1	61	1	1	0	1	G/C
CRC-0800	1	57	1	1	0	2	G/C
CRC-0801	1	51	0	1	0	2	G/G
CRC-0802	1	81	0	0	0	1	G/C
CRC-0803	1	64	0	0	0	1	G/C
CRC-0804	1	74	1	1	0	1	G/C
CRC-0805	2	54	0	0	0	2	G/C
CRC-0806	2	44	0	0	1	2	G/C
CRC-0807	2	84	0	0	1	1	G/C
CRC-0808	1	49	1	0	1	2	G/C
CRC-0809	2	48	0	0	0	1	G/C
CRC-0810	1	82	0	0	0	1	G/C
CRC-0811	2	74	0	0	0	2	C/C
CRC-0812	1	78	0	0	0	1	C/C
CRC-0813	1	83	0	0	1	2	C/C
CRC-0814	2	40	0	0	0	2	G/C
CRC-0815	2	40	0	0	0	2	C/C
CRC-0816	2	54	0	0	0	1	G/C
CRC-0817	1	64	1	0	0	2	G/C
CRC-0818	1	62	1	0	0	2	G/C
CRC-0819	1	64	1	1	0	2	C/C
CRC-0820	1	71	0	0	0	2	G/G
CRC-0821	1	72	0	0	0	2	C/C
CRC-0822	2	53	0	0	0	1	G/C
CRC-0823	1	75	1	0	0	2	G/G
CRC-0824	2	62	0	0	0	2	C/C
CRC-0825	1	74	1	0	0	1	C/C
CRC-0826	1	66	0	0	1	1	C/C
CRC-0827	1	63	0	0	1	1	?
CRC-0828	1	59	1	0	1	1	G/G
CRC-0829	2	60	0	0	1	2	G/C
CRC-0830	2	65	0	0	0	1	G/G
CRC-0831	2	64	0	0	0	2	C/C
CRC-0832	1	48	1	0	0	1	C/C
CRC-0833	1	52	1	0	1	1	G/C
CRC-0834	1	27	1	0	0	2	G/C
CRC-0835	1	45	0	0	1	2	C/C
CRC-0836	2	65	0	0	0	2	C/C
CRC-0837	1	44	1	1	1	2	G/C
CRC-0838	1	67	1	1	0	2	G/C
CRC-0839	1	65	1	1	0	2	C/C
CRC-0840	2	52	0	0	0	2	G/G
CRC-0841	2	48	0	0	0	1	G/C
CRC-0842	1	64	1	0	0	2	C/C
CRC-0843	2	46	0	0	0	1	G/C
CRC-0844	2	60	0	0	0	2	C/C
CRC-0845	2	35	0	0	0	1	C/C
CRC-0846	2	68	0	0	0	1	C/C
CRC-0847	1	70	1	1	1	1	C/C
CRC-0848	1	35	1	1	0	2	G/C
CRC-0849	2	66	0	0	1	1	G/G
CRC-0850	2	38	0	0	0	2	C/C

CRC-0851	1	48	0	1	0	2	G/C
CRC-0852	1	57	1	0	0	2	G/C
CRC-0853	1	68	0	0	0	1	G/C
CRC-0854	1	65	1	0	0	2	G/C
CRC-0855	2	64	0	0	0	2	G/C
CRC-0856	1	38	0	1	1	2	G/G
CRC-0857	2	63	0	0	0	1	G/C
CRC-0858	1	62	0	0	0	2	G/C
CRC-0859	1	50	1	0	1	1	G/C
CRC-0860	2	69	0	0	0	2	C/C
CRC-0861	1	44	0	0	0	2	C/C
CRC-0862	1	39	0	0	0	2	C/C
CRC-0863	1	50	1	1	1	1	G/C
CRC-0864	2	48	0	0	1	1	G/G
CRC-0865	2	59	0	0	0	1	G/C
CRC-0866	2	70	0	0	0	1	C/C
CRC-0867	2	48	0	0	1	1	G/C
CRC-0868	1	63	0	0	0	1	G/C
CRC-0869	1	49	0	0	1	1	G/C
CRC-0870	1	31	0	1	0	2	G/C
CRC-0871	1	45	1	0	0	1	G/C
CRC-0872	1	51	1	0	0	2	C/C
CRC-0873	2	52	0	0	0	1	G/C
CRC-0874	1	68	1	1	0	1	G/C
CRC-0875	1	56	1	1	0	2	G/C
CRC-0876	1	65	0	0	0	2	C/C
CRC-0877	1	63	0	0	1	1	G/C
CRC-0878	1	74	0	0	0	1	C/C
CRC-0879	2	62	0	0	1	1	G/C
CRC-0880	2	82	0	0	0	2	C/C
CRC-0881	1	77	0	0	1	1	C/C
CRC-0882	2	33	0	0	1	2	C/C
CRC-0883	1	51	0	0	0	1	G/C
CRC-0884	1	70	1	0	0	1	G/C
CRC-0885	2	76	0	0	0	2	C/C
CRC-0886	1	36	0	0	0	2	G/C
CRC-0887	2	42	0	0	0	2	C/C
CRC-0888	2	64	0	0	1	1	C/C
CRC-0889	1	61	0	1	1	1	C/C
CRC-0890	1	77	1	1	0	2	G/C
CRC-0891	1	53	0	0	0	2	C/C
CRC-0892	2	75	0	0	0	1	G/G
CRC-0893	1	61	1	0	1	2	C/C
CRC-0894	1	80	1	0	0	2	G/G
CRC-0895	1	64	0	0	0	2	G/G
CRC-0896	1	47	0	0	0	2	G/C
CRC-0897	1	77	0	0	1	2	G/G
CRC-0898	1	60	1	0	1	2	G/C
CRC-0899	2	79	0	0	0	2	C/C
CRC-0900	1	65	1	0	0	1	G/C
CRC-0901	1	43	0	0	0	2	G/C
CRC-0902	2	53	0	0	0	2	C/C
CRC-0903	1	52	1	1	0	1	C/C
CRC-0904	1	52	0	0	0	2	C/C
CRC-0905	1	66	0	0	0	2	G/C
CRC-0906	1	55	0	0	0	1	C/C
CRC-0907	2	50	0	0	0	2	G/C
CRC-0908	2	43	0	0	1	2	G/C
CRC-0909	1	65	0	1	0	1	C/C
CRC-0910	1	59	1	0	1	2	C/C
CRC-0911	1	46	0	0	0	2	G/C
CRC-0912	2	56	0	0	0	2	C/C
CRC-0913	1	71	0	0	0	2	C/C
CRC-0914	2	60	0	0	1	2	G/C
CRC-0915	1	46	0	0	1	2	C/C
CRC-0916	2	53	0	0	0	2	G/C

CRC-0917	2	64	0	0	0	2	C/C
CRC-0918	2	51	0	0	1	2	C/C
CRC-0919	1	53	0	0	0	1	C/C
CRC-0920	2	52	0	0	0	2	C/C
CRC-0921	1	67	0	0	0	1	G/C
CRC-0922	2	76	0	0	0	2	G/C
CRC-0923	2	57	0	0	0	2	C/C
CRC-0924	2	68	0	0	0	1	C/C
CRC-0925	1	51	0	0	0	2	G/C
CRC-0926	2	43	0	0	0	2	G/C
CRC-0927	2	67	0	0	0	1	C/C
CRC-0928	2	43	0	0	0	2	G/C
CRC-0929	2	47	0	0	0	1	G/G
CRC-0930	1	68	0	0	1	1	G/C
CRC-0931	1	69	0	0	0	2	G/C
CRC-0932	1	66	1	0	1	1	C/C
CRC-0933	1	86	0	0	1	1	G/G
CRC-0934	1	77	1	0	1	2	G/C
CRC-0935	1	62	1	1	0	1	C/C
CRC-0936	1	66	0	0	0	2	C/C
CRC-0937	1	63	1	0	1	2	G/C
CRC-0938	2	61	0	0	0	2	C/C
CRC-0939	2	44	0	0	0	1	G/G
CRC-0940	2	58	0	0	0	1	G/C
CRC-0941	2	53	0	0	1	1	C/C
CRC-0942	1	61	0	0	0	1	G/C
CRC-0943	1	71	0	0	0	2	C/C
CRC-0944	1	54	1	0	1	2	G/C
CRC-0945	2	67	0	0	0	2	G/G
CRC-0946	1	49	0	0	1	2	G/C
CRC-0947	1	58	0	0	0	1	G/C
CRC-0948	1	63	1	0	1	1	G/G
CRC-0949	2	75	0	0	0	1	G/C
CRC-0950	1	60	1	0	0	2	C/C
CRC-0951	1	56	0	0	1	2	G/C
CRC-0952	1	60	0	0	1	1	C/C
CRC-0953	2	72	0	0	0	2	G/C
CRC-0954	1	50	0	0	0	2	G/C
CRC-0955	1	64	1	0	1	2	G/G
CRC-0956	1	65	0	0	0	2	G/C
CRC-0957	1	62	0	0	0	1	G/C
CRC-0958	1	49	0	0	0	2	G/G
CRC-0959	1	42	0	0	0	2	G/G
CRC-0960	1	74	0	0	1	1	G/C
CRC-0961	1	49	0	0	0	1	C/C
CRC-0962	1	55	0	0	0	1	C/C
CRC-0963	1	55	1	0	0	2	G/C
CRC-0964	2	72	0	0	1	2	G/C
CRC-0965	2	66	0	0	1	2	C/C
CRC-0966	2	59	0	0	1	1	G/C
CRC-0967	1	45	1	0	0	1	G/C
CRC-0968	2	43	0	0	0	2	C/C
CRC-0969	1	52	1	0	1	2	C/C
CRC-0970	1	65	0	0	1	1	G/C
CRC-0971	2	78	0	0	0	2	C/C
CRC-0972	1	60	0	0	0	2	G/C
CRC-0973	1	72	1	0	0	2	G/C
CRC-0974	1	57	1	1	1	2	C/C
CRC-0975	2	62	0	0	0	2	C/C
CRC-0976	2	58	0	0	0	2	C/C
CRC-0977	2	48	0	0	1	2	C/C
CRC-0978	1	62	1	0	0	1	C/C
CRC-0979	1	64	0	0	0	1	G/C
CRC-0980	1	55	0	0	0	1	G/C
CRC-0981	2	51	0	0	0	1	G/C
CRC-0982	2	79	0	0	0	2	C/C

CRC-0983	2	78	0	0	0	2	C/C
CRC-0984	2	62	0	0	0	1	G/C
CRC-0985	2	50	0	0	0	2	G/C
CRC-0986	2	60	0	0	1	1	G/C
CRC-0987	2	58	0	0	0	1	G/C
CRC-0988	1	55	0	0	1	2	C/C
CRC-0989	1	28	1	0	0	1	C/C
CRC-0990	1	70	1	0	1	2	C/C
CRC-0991	2	81	0	0	1	1	C/C
CRC-0992	1	57	1	1	0	2	G/C
CRC-0993	1	55	1	0	1	2	C/C
CRC-0994	1	70	0	0	0	1	G/C
CRC-0995	1	65	0	0	1	1	C/C
CRC-0996	2	59	0	0	1	2	C/C
CRC-0997	2	68	0	0	1	2	G/C
CRC-0998	1	60	0	0	0	2	G/C
CRC-0999	1	72	0	0	0	2	G/C
CRC-1000	1	53	0	0	1	2	G/C
CRC-1001	1	52	1	0	1	2	G/C
CRC-1002	1	52	0	0	0	2	G/G
CRC-1003	1	50	0	0	1	2	C/C
Control-0001	1	55	0	0	1		G/G
Control-0002	2	53	0	0	1		G/C
Control-0003	1	59	0	0	1		G/C
Control-0004	1	74	0	0	1		G/C
Control-0005	2	54	0	0	0		G/C
Control-0006	2	70	0	0	1		C/C
Control-0007	1	65	1	0	0		G/C
Control-0008	1	84	0	0	0		C/C
Control-0009	1	62	0	0	0		C/C
Control-0010	2	77	0	0	1		G/C
Control-0011	2	56	0	0	0		G/G
Control-0012	1	63	0	0	0		G/C
Control-0013	1	51	0	0	1		C/C
Control-0014	2	61	0	0	0		G/C
Control-0015	1	57	0	0	1		G/C
Control-0016	1	81	0	0	1		G/C
Control-0017	1	79	0	0	1		G/C
Control-0018	1	53	1	0	0		G/C
Control-0019	1	65	0	0	0		G/G
Control-0020	1	56	0	1	1		G/C
Control-0021	1	64	1	1	1		G/G
Control-0022	1	62	0	0	1		C/C
Control-0023	1	72	1	0	0		G/C
Control-0024	1	66	0	0	1		G/G
Control-0025	1	29	1	0	1		G/G
Control-0026	2	71	0	0	0		C/C
Control-0027	1	38	0	0	1		C/C
Control-0028	1	71	0	0	1		G/C
Control-0029	1	60	1	0	1		C/C
Control-0030	2	53	0	0	1		C/C
Control-0031	1	58	0	0	0		G/C
Control-0032	2	52	0	0	0		G/C
Control-0033	1	67	1	0	0		C/C
Control-0034	2	69	0	0	1		G/C
Control-0035	1	80	0	0	1		G/C
Control-0036	1	77	0	0	1		C/C
Control-0037	2	38	0	0	0		G/C
Control-0038	1	28	0	0	0		G/C
Control-0039	2	65	0	0	1		C/C
Control-0040	2	52	0	0	0		C/C
Control-0041	1	66	0	0	0		G/C
Control-0042	2	46	0	0	0		G/G
Control-0043	1	56	0	0	1		G/C
Control-0044	2	68	0	0	1		C/C
Control-0045	1	59	0	0	0		G/C

Control-0046	1	62	0	0	1	C/C
Control-0047	1	71	0	0	0	G/C
Control-0048	1	60	1	1	0	G/C
Control-0049	1	56	1	0	0	C/C
Control-0050	1	73	1	0	0	G/G
Control-0051	1	60	0	0	0	G/C
Control-0052	2	72	0	0	0	G/C
Control-0053	2	61	0	0	0	G/C
Control-0054	2	66	0	0	0	G/C
Control-0055	1	61	1	0	0	C/C
Control-0056	1	71	1	0	0	C/C
Control-0057	1	77	0	0	0	C/C
Control-0058	1	64	1	0	0	C/C
Control-0059	2	54	0	0	0	G/C
Control-0060	1	63	0	0	0	C/C
Control-0061	2	56	0	0	0	G/G
Control-0062	1	63	1	0	0	C/C
Control-0063	2	71	0	0	1	G/C
Control-0064	1	72	0	0	0	G/C
Control-0065	1	46	0	0	0	G/G
Control-0066	1	62	1	0	0	C/C
Control-0067	2	59	0	0	0	C/C
Control-0068	2	79	0	0	0	G/C
Control-0069	2	60	0	0	1	G/G
Control-0070	2	52	0	0	1	C/C
Control-0071	1	56	1	0	0	G/C
Control-0072	1	59	1	0	0	G/C
Control-0073	1	61	0	0	1	C/C
Control-0074	1	63	0	0	1	C/C
Control-0075	1	75	1	0	1	C/C
Control-0076	1	72	0	0	0	G/C
Control-0077	1	64	0	1	0	C/C
Control-0078	2	67	0	0	1	C/C
Control-0079	1	58	1	0	0	C/C
Control-0080	1	70	1	1	1	G/G
Control-0081	1	58	0	0	0	C/C
Control-0082	1	78	0	0	1	G/C
Control-0083	2	61	0	0	0	G/C
Control-0084	1	67	0	0	1	G/C
Control-0085	1	41	1	0	1	G/C
Control-0086	2	69	0	0	0	G/G
Control-0087	1	62	0	0	0	G/C
Control-0088	1	69	0	0	0	C/C
Control-0089	1	57	0	0	0	C/C
Control-0090	2	51	0	0	1	G/C
Control-0091	1	55	1	0	0	G/C
Control-0092	2	37	0	0	0	C/C
Control-0093	1	65	1	1	0	G/C
Control-0094	1	57	0	0	1	G/C
Control-0095	2	49	0	0	0	G/C
Control-0096	2	46	0	0	0	G/G
Control-0097	1	65	0	0	0	G/C
Control-0098	2	64	0	0	1	G/C
Control-0099	2	65	0	0	0	G/C
Control-0100	1	69	0	0	0	C/C
Control-0101	1	78	0	0	1	C/C
Control-0102	1	43	0	0	0	G/G
Control-0103	1	64	0	0	0	C/C
Control-0104	2	56	0	0	1	G/C
Control-0105	2	78	0	0	1	G/C
Control-0106	2	52	0	0	1	C/C
Control-0107	2	57	0	0	0	C/C
Control-0108	2	80	0	0	1	C/C
Control-0109	2	73	0	0	0	G/C
Control-0110	2	67	0	0	0	G/G
Control-0111	2	53	0	0	0	G/C

Control-0112	1	48	1	0	1	C/C
Control-0113	1	57	1	0	0	C/C
Control-0114	1	62	1	0	1	G/C
Control-0115	1	69	1	1	0	G/C
Control-0116	2	53	0	0	0	G/C
Control-0117	1	75	0	0	1	G/G
Control-0118	2	49	0	0	1	G/C
Control-0119	2	61	0	0	0	G/C
Control-0120	2	62	0	0	1	C/C
Control-0121	1	53	1	0	1	G/C
Control-0122	1	62	0	0	1	G/C
Control-0123	1	63	0	0	0	G/C
Control-0124	1	33	0	0	0	G/G
Control-0125	1	81	0	0	1	G/G
Control-0126	2	59	0	0	1	C/C
Control-0127	2	56	0	0	0	C/C
Control-0128	1	69	0	0	1	G/C
Control-0129	1	58	0	0	0	G/C
Control-0130	1	60	1	0	0	C/C
Control-0131	1	59	0	0	1	C/C
Control-0132	1	76	1	0	0	C/C
Control-0133	1	52	0	0	0	G/G
Control-0134	2	34	0	0	0	C/C
Control-0135	1	60	1	0	1	C/C
Control-0136	1	76	1	0	0	G/C
Control-0137	1	87	1	1	0	C/C
Control-0138	1	81	0	0	0	G/C
Control-0139	2	50	0	0	1	G/C
Control-0140	1	57	0	0	0	G/G
Control-0141	1	51	0	0	0	C/C
Control-0142	1	76	0	0	0	C/C
Control-0143	1	64	0	0	0	G/C
Control-0144	1	60	0	0	0	C/C
Control-0145	2	65	0	0	1	G/G
Control-0146	1	55	0	0	1	G/C
Control-0147	2	70	0	0	0	C/C
Control-0148	1	38	1	0	0	C/C
Control-0149	1	64	0	0	1	G/G
Control-0150	1	62	0	0	0	G/G
Control-0151	1	54	1	0	0	C/C
Control-0152	1	64	0	0	0	C/C
Control-0153	1	56	0	0	0	G/G
Control-0154	2	60	0	0	1	C/C
Control-0155	1	64	0	0	0	G/C
Control-0156	1	78	0	0	1	C/C
Control-0157	1	58	0	0	0	C/C
Control-0158	1	46	0	0	0	G/G
Control-0159	2	72	0	0	0	G/C
Control-0160	1	21	1	0	0	G/C
Control-0161	1	44	1	0	1	C/C
Control-0162	2	41	0	0	0	G/G
Control-0163	1	46	1	0	1	C/C
Control-0164	2	67	0	0	0	C/C
Control-0165	2	65	0	0	0	G/G
Control-0166	2	66	0	0	1	G/C
Control-0167	1	61	1	0	0	C/C
Control-0168	1	52	0	0	0	C/C
Control-0169	2	67	0	0	1	G/G
Control-0170	1	60	0	0	1	G/G
Control-0171	1	56	1	0	0	C/C
Control-0172	1	60	0	0	1	C/C
Control-0173	1	57	0	0	1	C/C
Control-0174	2	65	0	0	0	G/G
Control-0175	2	70	0	0	0	G/C
Control-0176	2	69	0	0	1	G/C
Control-0177	1	74	0	0	0	G/C

Control-0178	1	63	1	0	1	G/C
Control-0179	1	53	1	0	0	C/C
Control-0180	2	25	0	0	0	G/C
Control-0181	2	79	0	0	0	G/G
Control-0182	2	43	0	0	0	C/C
Control-0183	1	62	1	0	1	C/C
Control-0184	2	74	0	0	1	C/C
Control-0185	2	73	0	0	0	G/C
Control-0186	2	62	0	0	0	C/C
Control-0187	1	74	1	0	0	G/C
Control-0188	1	58	0	0	0	C/C
Control-0189	2	51	0	0	0	G/C
Control-0190	1	61	0	0	1	C/C
Control-0191	1	66	0	0	1	C/C
Control-0192	1	74	0	0	0	G/C
Control-0193	1	55	0	0	1	G/C
Control-0194	1	56	0	0	0	G/C
Control-0195	1	43	0	0	1	C/C
Control-0196	2	63	0	0	1	C/C
Control-0197	2	61	0	0	1	G/C
Control-0198	1	59	1	0	1	C/C
Control-0199	1	60	1	0	0	C/C
Control-0200	1	77	0	0	1	G/C
Control-0201	1	79	0	0	1	C/C
Control-0202	2	63	0	0	1	G/C
Control-0203	2	39	0	0	1	G/C
Control-0204	1	42	0	0	1	G/C
Control-0205	1	68	0	1	0	G/C
Control-0206	1	58	0	0	0	C/C
Control-0207	1	54	0	0	0	G/G
Control-0208	1	57	1	0	1	G/C
Control-0209	1	66	0	0	0	C/C
Control-0210	1	74	0	0	1	C/C
Control-0211	2	50	0	0	0	G/C
Control-0212	1	68	0	0	1	G/G
Control-0213	1	60	0	0	1	C/C
Control-0214	1	44	1	0	1	C/C
Control-0215	2	59	0	0	1	G/C
Control-0216	1	73	0	0	1	G/C
Control-0217	1	75	1	0	1	C/C
Control-0218	1	65	0	0	0	C/C
Control-0219	1	62	0	0	0	G/C
Control-0220	1	57	0	0	0	C/C
Control-0221	1	46	0	0	1	G/C
Control-0222	1	47	1	0	1	C/C
Control-0223	2	60	0	0	1	G/C
Control-0224	1	59	0	0	0	G/C
Control-0225	2	52	0	0	0	G/C
Control-0226	1	52	0	0	1	C/C
Control-0227	1	76	0	0	1	G/C
Control-0228	1	75	0	0	1	C/C
Control-0229	2	51	0	0	1	C/C
Control-0230	1	66	0	0	1	C/C
Control-0231	1	47	1	0	1	G/C
Control-0232	2	61	0	0	0	C/C
Control-0233	1	44	0	0	0	G/G
Control-0234	2	62	0	0	1	G/C
Control-0235	1	40	0	0	1	G/C
Control-0236	1	50	0	1	1	C/C
Control-0237	2	59	0	0	0	C/C
Control-0238	1	48	0	0	1	C/C
Control-0239	1	74	0	1	1	C/C
Control-0240	1	57	0	0	0	C/C
Control-0241	1	69	0	0	1	C/C
Control-0242	1	62	0	0	1	C/C
Control-0243	2	57	0	0	1	C/C

Control-0244	1	37	0	0	0	G/C
Control-0245	1	64	0	0	1	G/C
Control-0246	1	64	0	0	0	G/G
Control-0247	1	45	0	0	1	G/G
Control-0248	1	77	0	0	0	G/C
Control-0249	1	60	0	0	0	C/C
Control-0250	1	68	0	0	0	G/G
Control-0251	1	49	1	1	0	G/C
Control-0252	1	49	0	0	0	C/C
Control-0253	1	43	0	0	1	G/C
Control-0254	1	81	0	0	1	G/C
Control-0255	1	46	0	0	1	G/C
Control-0256	2	55	0	0	1	C/C
Control-0257	1	79	1	1	0	C/C
Control-0258	1	71	1	1	0	G/C
Control-0259	1	64	0	0	0	G/C
Control-0260	2	57	0	0	0	C/C
Control-0261	1	58	1	0	1	C/C
Control-0262	1	74	0	0	0	G/C
Control-0263	1	41	0	0	1	C/C
Control-0264	2	54	0	0	1	G/C
Control-0265	1	59	0	1	0	C/C
Control-0266	1	70	0	0	0	C/C
Control-0267	1	55	0	0	1	G/C
Control-0268	1	35	0	0	0	G/C
Control-0269	1	58	0	0	0	C/C
Control-0270	1	59	0	0	1	G/G
Control-0271	1	60	0	0	0	C/C
Control-0272	1	58	0	0	0	G/C
Control-0273	2	60	0	0	0	C/C
Control-0274	2	61	0	0	0	C/C
Control-0275	1	86	0	0	1	G/C
Control-0276	2	67	0	0	0	C/C
Control-0277	1	59	1	0	1	C/C
Control-0278	2	55	0	0	0	C/C
Control-0279	1	66	0	0	0	G/C
Control-0280	1	64	0	0	1	C/C
Control-0281	1	71	0	0	0	C/C
Control-0282	1	56	1	0	1	C/C
Control-0283	2	61	0	0	1	C/C
Control-0284	1	73	1	0	0	G/C
Control-0285	1	60	1	0	0	C/C
Control-0286	2	66	0	0	0	G/C
Control-0287	2	63	0	0	1	G/C
Control-0288	1	65	1	0	1	G/G
Control-0289	1	60	0	0	0	C/C
Control-0290	2	51	0	0	0	C/C
Control-0291	1	57	0	0	1	G/C
Control-0292	2	79	0	0	1	C/C
Control-0293	1	55	1	1	0	G/C
Control-0294	1	51	0	0	1	C/C
Control-0295	2	66	0	0	0	C/C
Control-0296	1	53	1	0	0	G/G
Control-0297	1	61	0	0	1	G/C
Control-0298	2	79	0	0	0	G/C
Control-0299	1	62	0	0	1	C/C
Control-0300	1	60	1	0	1	G/C
Control-0301	1	71	1	1	1	G/C
Control-0302	1	67	0	0	0	C/C
Control-0303	2	69	0	0	0	G/C
Control-0304	1	62	0	0	0	G/C
Control-0305	1	61	1	0	0	G/C
Control-0306	1	62	1	0	0	G/C
Control-0307	2	70	0	0	1	C/C
Control-0308	2	56	0	0	1	G/C
Control-0309	2	68	0	0	1	C/C

Control-0310	2	53	0	0	1	G/C
Control-0311	2	56	0	0	1	G/G
Control-0312	2	48	0	0	0	G/C
Control-0313	1	43	1	0	0	G/C
Control-0314	1	61	0	0	1	G/C
Control-0315	1	67	0	0	1	G/C
Control-0316	1	57	0	0	0	C/C
Control-0317	1	39	0	0	1	G/C
Control-0318	1	87	0	0	1	G/G
Control-0319	1	63	0	0	0	G/C
Control-0320	1	74	1	0	0	C/C
Control-0321	1	76	0	0	1	G/C
Control-0322	1	67	0	0	0	C/C
Control-0323	1	61	0	0	0	C/C
Control-0324	1	74	0	1	0	G/C
Control-0325	2	65	0	0	1	G/C
Control-0326	1	71	1	0	0	C/C
Control-0327	1	59	1	0	0	G/C
Control-0328	1	69	1	0	0	G/G
Control-0329	2	78	0	0	0	C/C
Control-0330	1	59	0	0	1	G/C
Control-0331	1	63	0	0	1	G/C
Control-0332	1	82	1	0	0	C/C
Control-0333	2	63	0	0	1	G/C
Control-0334	1	71	0	0	1	G/G
Control-0335	1	64	0	1	0	G/G
Control-0336	1	56	0	0	0	C/C
Control-0337	2	72	0	0	1	C/C
Control-0338	1	62	1	0	0	G/G
Control-0339	2	51	0	0	0	G/G
Control-0340	1	87	0	0	0	G/C
Control-0341	1	58	1	0	0	G/C
Control-0342	1	60	0	0	0	G/C
Control-0343	2	66	0	0	0	G/C
Control-0344	2	70	0	0	0	C/C
Control-0345	2	81	0	0	1	C/C
Control-0346	1	57	0	0	1	C/C
Control-0347	2	61	0	0	0	C/C
Control-0348	2	59	0	0	0	C/C
Control-0349	1	79	0	0	0	G/C
Control-0350	2	54	0	0	0	C/C
Control-0351	1	64	0	0	0	G/C
Control-0352	2	48	0	0	1	G/C
Control-0353	1	74	1	0	1	C/C
Control-0354	1	63	1	0	0	G/C
Control-0355	2	69	0	0	0	G/C
Control-0356	2	74	0	0	1	G/C
Control-0357	1	59	0	0	1	G/G
Control-0358	2	63	0	0	1	C/C
Control-0359	1	73	0	0	1	C/C
Control-0360	2	68	0	0	0	G/C
Control-0361	1	76	0	0	0	G/C
Control-0362	1	65	0	0	0	G/G
Control-0363	1	73	0	0	1	G/G
Control-0364	2	59	0	0	0	G/G
Control-0365	1	65	1	0	0	G/C
Control-0366	1	66	0	0	0	C/C
Control-0367	2	67	0	0	0	C/C
Control-0368	2	55	0	0	0	C/C
Control-0369	1	78	0	0	1	C/C
Control-0370	1	71	0	0	1	G/C
Control-0371	2	67	0	0	0	C/C
Control-0372	1	53	0	0	1	C/C
Control-0373	2	67	0	0	1	C/C
Control-0374	2	68	0	0	1	G/C
Control-0375	2	82	0	0	0	G/G

Control-0376	1	55	1	0	0	G/C
Control-0377	2	63	0	0	1	G/C
Control-0378	1	38	0	0	1	C/C
Control-0379	2	72	0	0	0	G/C
Control-0380	1	56	1	0	1	G/C
Control-0381	1	80	0	0	0	G/C
Control-0382	1	56	0	0	1	G/C
Control-0383	1	65	0	0	0	G/C
Control-0384	1	59	0	0	1	G/C
Control-0385	1	72	0	0	0	C/C
Control-0386	1	59	1	0	1	C/C
Control-0387	1	77	1	0	0	G/C
Control-0388	1	60	0	0	0	C/C
Control-0389	1	56	1	0	0	G/C
Control-0390	1	49	0	0	0	C/C
Control-0391	1	62	0	0	0	C/C
Control-0392	2	68	0	0	0	C/C
Control-0393	1	70	1	0	1	C/C
Control-0394	2	65	0	0	1	G/C
Control-0395	2	77	0	0	1	G/C
Control-0396	1	49	1	0	0	G/C
Control-0397	1	71	1	0	1	C/C
Control-0398	1	52	0	0	0	G/C
Control-0399	1	47	0	0	0	C/C
Control-0400	1	48	0	0	1	C/C
Control-0401	2	46	0	0	0	C/C
Control-0402	2	51	0	0	0	C/C
Control-0403	1	67	1	0	1	G/C
Control-0404	1	78	0	0	0	C/C
Control-0405	1	48	1	0	0	C/C
Control-0406	2	56	0	0	0	G/C
Control-0407	2	51	0	0	1	G/G
Control-0408	2	63	0	0	1	G/C
Control-0409	2	54	0	0	1	C/C
Control-0410	2	52	0	0	1	G/C
Control-0411	1	52	0	0	1	G/C
Control-0412	1	58	0	0	0	C/C
Control-0413	1	78	0	0	0	G/C
Control-0414	1	51	0	0	0	G/C
Control-0415	2	68	0	0	1	G/C
Control-0416	1	52	0	0	0	C/C
Control-0417	2	51	0	0	1	C/C
Control-0418	2	66	0	0	1	C/C
Control-0419	2	62	0	0	0	C/C
Control-0420	1	66	0	0	0	G/C
Control-0421	1	65	0	0	1	C/C
Control-0422	2	60	0	0	0	G/C
Control-0423	1	48	0	0	1	G/C
Control-0424	2	53	0	0	1	G/C
Control-0425	1	70	1	0	1	G/C
Control-0426	2	60	0	0	1	G/C
Control-0427	2	63	0	0	0	G/C
Control-0428	2	61	0	0	1	G/C
Control-0429	1	78	0	0	0	G/G
Control-0430	1	55	0	0	0	G/C
Control-0431	1	69	0	0	1	C/C
Control-0432	1	67	0	0	1	C/C
Control-0433	1	62	0	0	0	G/C
Control-0434	2	49	0	0	0	C/C
Control-0435	1	55	0	0	1	C/C
Control-0436	1	62	1	0	0	G/C
Control-0437	1	48	1	0	1	G/C
Control-0438	1	49	1	0	1	G/C
Control-0439	1	61	0	0	1	C/C
Control-0440	2	77	0	0	1	C/C
Control-0441	2	53	0	0	0	C/C

Control-0442	1	69	1	0	0	C/C
Control-0443	2	70	0	0	1	G/C
Control-0444	1	52	0	0	0	G/C
Control-0445	2	61	0	0	0	C/C
Control-0446	2	67	0	0	0	C/C
Control-0447	1	30	0	0	0	G/G
Control-0448	1	58	0	0	0	C/C
Control-0449	2	61	0	0	1	C/C
Control-0450	1	75	0	0	0	G/C
Control-0451	1	54	0	0	0	C/C
Control-0452	2	42	0	0	0	C/C
Control-0453	2	61	0	0	0	G/C
Control-0454	1	57	0	0	0	G/G
Control-0455	1	50	1	0	1	C/C
Control-0456	2	66	0	0	0	C/C
Control-0457	1	55	1	0	1	G/G
Control-0458	2	52	0	0	1	G/C
Control-0459	1	41	0	0	0	C/C
Control-0460	2	63	0	0	1	G/C
Control-0461	1	59	0	0	1	G/C
Control-0462	1	74	1	1	0	G/C
Control-0463	1	68	0	0	0	G/C
Control-0464	1	63	1	1	1	G/C
Control-0465	1	60	1	0	0	C/C
Control-0466	1	59	0	0	1	G/C
Control-0467	2	57	0	0	1	C/C
Control-0468	1	60	1	0	1	C/C
Control-0469	2	42	0	0	0	G/C
Control-0470	1	54	1	0	0	G/C
Control-0471	2	42	0	0	1	C/C
Control-0472	1	51	0	0	0	G/C
Control-0473	1	50	0	0	1	G/C
Control-0474	1	58	0	0	1	C/C
Control-0475	2	65	1	0	1	C/C
Control-0476	1	51	0	0	0	G/C
Control-0477	1	64	0	0	1	G/C
Control-0478	1	27	0	0	0	C/C
Control-0479	1	63	1	0	0	G/C
Control-0480	1	53	0	0	0	G/C
Control-0481	2	64	0	0	0	C/C
Control-0482	1	55	1	0	0	C/C
Control-0483	1	39	1	0	0	C/C
Control-0484	1	69	0	0	1	C/C
Control-0485	1	50	1	0	0	C/C
Control-0486	1	76	0	0	0	G/C
Control-0487	1	79	1	0	0	C/C
Control-0488	1	52	0	0	0	C/C
Control-0489	2	58	0	0	1	G/C
Control-0490	1	43	0	0	0	G/C
Control-0491	2	53	0	0	0	C/C
Control-0492	1	78	0	0	0	G/C
Control-0493	1	63	1	0	0	G/C
Control-0494	1	57	1	0	1	G/C
Control-0495	2	60	0	0	0	G/G
Control-0496	1	67	0	0	1	G/C
Control-0497	1	66	0	0	1	G/C
Control-0498	1	62	0	0	1	G/G
Control-0499	2	61	0	0	1	G/G
Control-0500	1	61	0	0	1	G/C
Control-0501	1	66	0	0	1	G/G
Control-0502	1	52	0	0	1	C/C
Control-0503	1	60	1	0	0	C/C
Control-0504	1	72	1	1	0	G/C
Control-0505	1	70	0	0	0	G/C
Control-0506	1	68	0	0	0	C/C
Control-0507	1	71	1	0	0	G/C

Control-0508	2	61	0	0	1	C/C
Control-0509	1	69	0	0	1	G/C
Control-0510	2	52	0	0	1	G/G
Control-0511	1	41	0	0	0	C/C
Control-0512	1	52	0	0	0	G/C
Control-0513	2	44	0	0	0	G/C
Control-0514	2	50	0	0	0	G/C
Control-0515	1	63	0	0	1	G/C
Control-0516	1	59	1	0	1	G/G
Control-0517	1	44	0	0	1	C/C
Control-0518	2	66	0	0	0	C/C
Control-0519	1	59	1	1	0	C/C
Control-0520	1	61	1	0	1	G/C
Control-0521	2	53	0	0	1	C/C
Control-0522	1	66	1	0	1	G/C
Control-0523	2	54	0	0	0	C/C
Control-0524	2	51	0	0	1	G/C
Control-0525	2	61	0	0	0	G/G
Control-0526	1	56	0	0	1	G/C
Control-0527	1	52	0	0	1	C/C
Control-0528	2	49	0	0	1	C/C
Control-0529	2	48	0	0	0	C/C
Control-0530	2	45	0	0	1	G/C
Control-0531	2	47	0	0	0	C/C
Control-0532	2	65	0	0	1	C/C
Control-0533	1	73	1	0	0	G/C
Control-0534	2	59	0	0	0	C/C
Control-0535	1	59	0	0	0	G/C
Control-0536	1	67	0	0	0	C/C
Control-0537	1	59	0	0	1	C/C
Control-0538	1	71	0	0	0	C/C
Control-0539	1	59	0	0	1	G/C
Control-0540	1	47	1	0	1	G/C
Control-0541	2	68	0	0	0	G/G
Control-0542	1	64	0	1	0	G/C
Control-0543	2	50	0	0	1	G/C
Control-0544	2	50	0	0	0	G/C
Control-0545	1	70	0	0	0	C/C
Control-0546	2	73	0	0	1	C/C
Control-0547	2	63	0	0	0	G/C
Control-0548	1	53	0	0	0	G/C
Control-0549	2	59	0	0	0	G/C
Control-0550	1	58	0	0	0	C/C
Control-0551	2	53	0	0	1	C/C
Control-0552	2	65	0	0	1	C/C
Control-0553	2	73	0	0	1	G/C
Control-0554	2	56	0	0	1	G/C
Control-0555	1	59	0	0	0	G/C
Control-0556	2	51	0	0	1	C/C
Control-0557	1	62	0	0	1	G/C
Control-0558	1	55	0	0	0	G/C
Control-0559	2	59	0	0	1	G/C
Control-0560	1	62	0	0	1	C/C
Control-0561	1	69	0	0	0	G/C
Control-0562	1	29	0	0	1	G/G
Control-0563	1	59	0	0	0	G/C
Control-0564	1	70	0	0	1	C/C
Control-0565	1	52	1	0	0	G/C
Control-0566	1	60	0	0	1	G/C
Control-0567	2	61	0	0	0	C/C
Control-0568	1	69	0	0	1	G/G
Control-0569	2	77	0	0	1	G/C
Control-0570	2	72	0	0	0	G/C
Control-0571	2	77	0	0	0	G/C
Control-0572	2	69	0	0	1	C/C
Control-0573	1	59	0	0	1	C/C

Control-0574	2	63	0	0	1	C/C
Control-0575	2	66	0	0	0	G/C
Control-0576	2	59	0	0	1	C/C
Control-0577	1	57	0	0	1	C/C
Control-0578	2	72	0	0	1	G/C
Control-0579	1	63	0	0	0	C/C
Control-0580	2	68	0	0	1	G/C
Control-0581	2	79	0	0	1	G/C
Control-0582	1	66	0	0	1	G/G
Control-0583	1	61	1	0	1	C/C
Control-0584	1	53	0	0	1	G/C
Control-0585	2	58	0	0	0	G/C
Control-0586	2	78	0	0	1	C/C
Control-0587	1	62	0	0	1	G/C
Control-0588	2	66	0	0	1	G/C
Control-0589	1	69	1	0	0	G/C
Control-0590	1	73	0	0	0	G/C
Control-0591	1	57	1	0	0	G/C
Control-0592	1	57	1	0	1	C/C
Control-0593	1	46	1	1	1	G/C
Control-0594	2	73	0	0	1	C/C
Control-0595	1	60	1	1	0	G/G
Control-0596	2	65	0	0	0	G/C
Control-0597	1	66	1	0	0	G/C
Control-0598	1	62	0	0	0	C/C
Control-0599	2	71	0	0	0	G/C
Control-0600	1	54	1	0	0	C/C
Control-0601	1	53	0	0	0	C/C
Control-0602	2	64	0	0	1	G/C
Control-0603	1	64	1	1	1	G/C
Control-0604	1	55	0	0	1	C/C
Control-0605	2	60	0	0	0	G/C
Control-0606	1	59	1	0	1	G/C
Control-0607	1	63	1	0	0	C/C
Control-0608	1	62	0	1	1	C/C
Control-0609	1	66	1	0	1	C/C
Control-0610	1	53	0	0	1	G/C
Control-0611	1	52	0	0	0	G/C
Control-0612	1	67	0	0	0	G/C
Control-0613	1	54	1	0	1	G/C
Control-0614	1	55	0	0	0	C/C
Control-0615	2	69	0	0	1	G/C
Control-0616	2	74	0	0	0	C/C
Control-0617	2	76	0	0	1	G/C
Control-0618	1	65	0	1	0	C/C
Control-0619	1	58	0	1	1	C/C
Control-0620	1	68	0	0	1	G/C
Control-0621	1	51	1	0	1	C/C
Control-0622	1	48	1	0	1	C/C
Control-0623	2	54	0	0	0	G/C
Control-0624	2	60	0	0	1	G/G
Control-0625	2	64	0	0	0	C/C
Control-0626	1	66	0	0	1	G/C
Control-0627	1	68	0	0	0	G/G
Control-0628	1	59	1	1	1	G/C
Control-0629	1	67	1	0	0	G/C
Control-0630	2	59	0	0	0	G/C
Control-0631	1	73	0	0	0	G/C
Control-0632	1	56	1	0	0	G/C
Control-0633	1	60	0	0	0	C/C
Control-0634	1	51	0	0	0	C/C
Control-0635	1	58	1	0	0	C/C
Control-0636	2	71	0	0	0	G/G
Control-0637	2	58	0	0	0	G/C
Control-0638	1	68	0	0	1	G/C
Control-0639	2	61	0	0	0	G/C

Control-0640	1	68	0	0	0	G/C
Control-0641	1	62	1	1	1	C/C
Control-0642	1	69	0	0	0	C/C
Control-0643	1	54	0	0	0	G/G
Control-0644	1	58	0	0	1	C/C
Control-0645	2	60	0	0	1	G/C
Control-0646	1	69	1	1	0	G/C
Control-0647	1	59	1	0	1	G/C
Control-0648	2	59	0	0	1	G/G
Control-0649	1	64	0	0	1	C/C
Control-0650	1	58	1	0	0	G/C
Control-0651	1	60	0	0	1	G/C
Control-0652	1	60	0	1	1	C/C
Control-0653	2	50	0	0	0	G/C
Control-0654	1	54	1	1	1	C/C
Control-0655	1	58	0	0	1	G/G
Control-0656	1	68	1	0	1	G/C
Control-0657	1	57	0	0	0	G/G
Control-0658	2	71	0	0	0	G/G
Control-0659	1	62	0	0	1	C/C
Control-0660	1	66	0	0	0	G/C
Control-0661	1	65	0	0	1	G/C
Control-0662	1	61	0	0	1	C/C
Control-0663	2	61	0	0	0	C/C
Control-0664	2	57	0	0	0	G/C
Control-0665	1	56	1	1	1	C/C
Control-0666	2	71	0	0	1	G/G
Control-0667	2	59	0	0	1	G/G
Control-0668	2	61	0	0	1	G/G
Control-0669	2	54	0	1	0	G/C
Control-0670	1	65	0	0	1	C/C
Control-0671	1	64	1	0	0	C/C
Control-0672	2	66	0	0	1	G/C
Control-0673	1	56	1	1	0	G/C
Control-0674	1	51	1	0	1	G/C
Control-0675	1	54	0	0	1	G/G
Control-0676	1	75	0	0	1	G/C
Control-0677	1	59	1	1	0	G/C
Control-0678	1	57	1	0	1	C/C
Control-0679	1	66	0	1	1	C/C
Control-0680	1	73	0	0	1	G/G
Control-0681	1	63	0	1	0	G/C
Control-0682	2	77	0	0	0	C/C
Control-0683	1	52	1	1	0	G/C
Control-0684	2	65	0	0	0	G/C
Control-0685	1	59	1	0	0	G/C
Control-0686	1	67	0	0	0	C/C
Control-0687	1	64	0	1	1	C/C
Control-0688	1	57	1	0	1	C/C
Control-0689	1	52	0	0	1	G/C
Control-0690	1	73	0	0	0	G/G
Control-0691	1	51	0	0	1	G/G
Control-0692	1	68	1	1	1	G/C
Control-0693	1	63	0	0	0	C/C
Control-0694	2	70	0	0	0	G/C
Control-0695	1	63	1	0	1	G/C
Control-0696	2	68	0	0	1	G/G
Control-0697	2	55	0	0	1	C/C
Control-0698	1	55	1	1	1	G/C
Control-0699	1	68	1	1	0	C/C
Control-0700	1	64	1	1	0	G/G
Control-0701	1	60	0	0	0	C/C
Control-0702	1	50	0	0	0	G/G
Control-0703	1	51	0	1	1	G/C
Control-0704	1	58	1	1	0	G/C
Control-0705	1	57	0	0	0	C/C

Control-0706	1	61	1	1	1	C/C
Control-0707	1	63	1	0	0	C/C
Control-0708	2	70	0	0	1	G/C
Control-0709	1	60	1	0	0	C/C
Control-0710	2	66	0	0	1	G/C
Control-0711	2	63	0	0	0	C/C
Control-0712	2	59	0	0	1	G/C
Control-0713	1	59	1	1	0	G/C
Control-0714	1	61	1	1	0	G/C
Control-0715	1	50	0	1	1	C/C
Control-0716	2	50	0	0	1	G/C
Control-0717	1	65	0	0	1	C/C
Control-0718	1	69	0	0	1	C/C
Control-0719	2	73	0	0	1	G/G
Control-0720	1	68	0	0	0	G/C
Control-0721	2	56	0	0	0	G/C
Control-0722	1	65	1	1	0	G/C
Control-0723	1	55	0	0	0	G/C
Control-0724	2	68	0	0	1	G/C
Control-0725	2	59	0	0	1	C/C
Control-0726	1	59	0	0	0	G/C
Control-0727	1	59	1	0	0	G/G
Control-0728	1	56	0	0	1	G/C
Control-0729	2	63	0	0	1	G/G
Control-0730	2	71	0	0	1	G/C
Control-0731	1	52	1	0	1	C/C
Control-0732	2	68	0	0	1	C/C
Control-0733	2	53	0	0	1	G/C
Control-0734	1	61	1	1	0	C/C
Control-0735	2	72	0	0	1	G/C
Control-0736	2	68	0	0	0	G/G
Control-0737	1	61	0	0	0	G/C
Control-0738	1	61	0	1	1	G/C
Control-0739	1	56	0	0	0	G/C
Control-0740	1	64	1	1	0	G/C
Control-0741	2	53	0	0	1	G/G
Control-0742	1	52	0	0	1	C/C
Control-0743	1	52	1	0	1	G/C
Control-0744	2	82	0	0	0	G/G
Control-0745	1	66	0	0	0	C/C
Control-0746	2	57	0	0	0	C/C
Control-0747	1	56	1	1	0	G/G
Control-0748	2	62	0	0	0	G/C
Control-0749	1	74	0	0	1	C/C
Control-0750	2	70	0	0	0	G/C
Control-0751	1	73	0	0	0	G/C
Control-0752	2	61	0	0	0	C/C
Control-0753	1	73	0	0	1	G/G
Control-0754	2	57	0	0	0	C/C
Control-0755	2	63	0	0	1	C/C
Control-0756	2	58	0	0	1	C/C
Control-0757	1	66	0	0	0	G/C
Control-0758	1	67	1	0	1	G/G
Control-0759	1	65	1	1	1	G/C
Control-0760	1	50	0	0	0	G/G
Control-0761	2	55	0	0	1	G/C
Control-0762	1	74	0	0	1	G/C
Control-0763	1	56	0	0	1	G/C
Control-0764	2	59	0	0	0	G/C
Control-0765	1	65	1	1	0	G/C
Control-0766	2	47	0	0	1	G/C
Control-0767	1	58	1	1	0	C/C
Control-0768	1	60	1	1	0	G/C
Control-0769	1	69	0	0	1	C/C
Control-0770	2	62	0	0	0	C/C
Control-0771	1	74	1	1	1	G/C

Control-0772	2	61	0	0	1	G/G
Control-0773	2	69	0	0	1	G/C
Control-0774	1	70	1	0	1	G/C
Control-0775	2	53	0	0	0	G/C
Control-0776	1	58	0	0	1	G/C
Control-0777	2	61	0	0	0	C/C
Control-0778	1	68	0	0	0	G/C
Control-0779	1	68	0	0	0	C/C
Control-0780	1	69	0	0	1	C/C
Control-0781	1	61	0	0	0	C/C
Control-0782	2	53	0	0	0	C/C
Control-0783	1	69	0	0	1	G/C
Control-0784	2	45	0	0	1	C/C
Control-0785	1	55	1	1	0	G/C
Control-0786	2	52	0	0	1	G/C
Control-0787	1	67	0	0	1	G/C
Control-0788	1	53	1	0	1	G/C
Control-0789	2	59	0	0	1	C/C
Control-0790	1	49	1	1	0	C/C
Control-0791	2	59	0	0	1	G/C
Control-0792	1	55	1	0	1	G/C
Control-0793	1	62	0	0	1	G/C
Control-0794	2	63	0	0	1	G/C
Control-0795	1	57	0	1	0	G/C
Control-0796	1	53	0	0	0	G/C
Control-0797	2	68	0	0	0	G/C
Control-0798	2	79	0	0	0	G/G
Control-0799	1	45	0	0	1	G/C
Control-0800	1	72	0	0	0	G/C
Control-0801	1	67	1	1	0	C/C
Control-0802	1	52	0	0	1	C/C
Control-0803	2	47	0	0	0	G/C
Control-0804	1	53	0	0	1	C/C
Control-0805	2	56	0	0	0	G/G
Control-0806	1	70	1	0	1	G/G
Control-0807	1	70	0	0	1	G/C
Control-0808	2	53	0	0	0	G/C
Control-0809	1	52	0	1	0	C/C
Control-0810	1	63	0	0	0	G/C
Control-0811	2	67	0	0	1	C/C
Control-0812	2	48	0	0	1	C/C
Control-0813	1	61	0	1	0	G/C
Control-0814	2	71	0	0	0	G/C
Control-0815	1	44	0	0	1	G/C
Control-0816	1	69	1	0	0	C/C
Control-0817	1	59	0	0	0	G/G
Control-0818	1	70	0	0	0	G/C
Control-0819	1	68	0	0	0	G/G
Control-0820	1	52	0	0	1	G/G
Control-0821	2	65	0	0	1	C/C
Control-0822	2	48	0	0	0	C/C
Control-0823	2	59	0	0	0	C/C
Control-0824	1	69	1	1	0	G/C
Control-0825	1	50	0	0	0	G/C
Control-0826	1	70	0	0	0	G/C
Control-0827	2	53	0	0	1	C/C
Control-0828	1	70	1	1	0	G/G
Control-0829	1	71	0	0	0	G/C
Control-0830	1	54	0	0	1	C/C
Control-0831	1	56	0	0	1	C/C
Control-0832	2	58	0	0	1	C/C
Control-0833	2	72	0	0	0	G/C
Control-0834	1	60	0	0	0	G/C
Control-0835	2	73	0	0	1	G/C
Control-0836	1	60	1	0	0	G/G
Control-0837	2	57	0	0	1	C/C

Control-0838	1	55	0	0	0	C/C
Control-0839	2	68	0	0	0	C/C
Control-0840	2	61	0	0	1	G/G
Control-0841	1	45	1	1	0	G/C
Control-0842	2	64	0	0	0	G/C
Control-0843	2	66	0	0	0	G/C
Control-0844	2	67	0	0	0	G/C
Control-0845	2	84	0	0	0	G/G
Control-0846	2	51	0	0	1	G/G
Control-0847	1	69	0	0	0	C/C
Control-0848	2	67	0	0	1	G/G
Control-0849	1	73	1	0	0	G/G
Control-0850	1	75	0	0	1	G/C
Control-0851	1	51	1	0	0	C/C
Control-0852	1	65	0	0	0	G/C
Control-0853	2	61	0	0	1	G/G
Control-0854	2	65	0	0	0	G/C
Control-0855	2	70	0	0	1	G/C
Control-0856	2	86	0	0	0	C/C
Control-0857	1	65	0	0	1	C/C
Control-0858	1	60	1	0	1	C/C
Control-0859	1	58	1	1	1	G/C
Control-0860	2	71	0	0	0	G/C
Control-0861	2	67	0	0	0	G/C
Control-0862	2	66	0	0	1	C/C
Control-0863	1	68	0	0	1	G/C
Control-0864	1	43	0	0	1	G/G
Control-0865	1	70	0	0	0	C/C
Control-0866	2	47	0	0	1	G/C
Control-0867	2	52	0	0	1	G/C
Control-0868	1	52	0	0	1	C/C
Control-0869	2	61	0	0	0	G/C
Control-0870	2	66	0	0	0	G/C
Control-0871	2	59	0	0	0	G/C
Control-0872	1	63	1	0	1	G/G
Control-0873	1	59	1	1	0	G/C
Control-0874	2	56	0	0	1	G/C
Control-0875	2	69	0	0	1	C/C
Control-0876	1	58	1	1	1	G/C
Control-0877	1	49	1	1	1	G/C
Control-0878	2	60	0	0	1	C/C
Control-0879	1	51	1	1	0	G/C
Control-0880	2	51	0	0	1	G/G
Control-0881	1	46	1	0	1	C/C
Control-0882	1	64	1	0	0	G/C
Control-0883	1	72	1	1	0	G/C
Control-0884	2	83	0	0	0	C/C
Control-0885	2	46	0	0	0	C/C
Control-0886	2	56	0	0	0	G/C
Control-0887	2	72	0	0	1	C/C
Control-0888	1	53	0	0	1	G/C
Control-0889	1	68	1	1	0	C/C
Control-0890	1	69	1	1	0	G/G
Control-0891	2	66	0	0	1	C/C
Control-0892	2	54	0	0	1	C/C
Control-0893	1	53	0	0	0	G/C
Control-0894	1	65	1	0	1	G/C
Control-0895	2	60	0	0	0	G/C
Control-0896	2	48	0	0	0	C/C
Control-0897	1	52	0	0	0	G/C
Control-0898	2	62	0	0	0	G/C
Control-0899	2	61	0	0	0	C/C
Control-0900	1	56	0	0	1	C/C
Control-0901	1	57	0	0	1	G/C
Control-0902	1	61	1	1	0	G/G
Control-0903	2	65	0	0	0	C/C

Control-0904	1	69	0	0	0	C/C
Control-0905	2	68	0	0	0	G/G
Control-0906	1	60	1	0	0	C/C
Control-0907	1	65	0	0	1	G/C
Control-0908	2	56	0	0	0	G/C
Control-0909	1	50	0	1	0	C/C
Control-0910	1	49	0	0	0	G/C
Control-0911	2	47	0	0	1	G/C
Control-0912	2	71	0	0	1	C/C
Control-0913	1	71	0	0	1	G/G
Control-0914	1	69	0	0	0	G/G
Control-0915	1	74	0	0	0	C/C
Control-0916	2	64	0	0	0	G/C
Control-0917	2	72	0	0	0	G/C
Control-0918	1	70	0	0	1	G/C
Control-0919	1	71	1	1	1	G/C
Control-0920	2	77	0	0	1	G/G
Control-0921	2	61	0	0	1	G/G
Control-0922	2	60	0	0	0	G/G
Control-0923	2	66	0	0	1	C/C
Control-0924	1	72	1	0	1	C/C
Control-0925	2	77	0	0	0	C/C
Control-0926	1	69	1	0	0	G/C
Control-0927	2	75	0	0	1	C/C
Control-0928	2	43	0	0	1	G/G
Control-0929	1	75	1	1	0	C/C
Control-0930	1	78	0	0	1	C/C
Control-0931	2	66	0	0	1	G/G
Control-0932	1	58	0	0	0	C/C
Control-0933	1	67	0	0	1	G/G
Control-0934	1	58	0	1	1	G/C
Control-0935	1	62	0	0	1	G/G
Control-0936	1	67	0	0	0	C/C
Control-0937	1	67	1	1	0	C/C
Control-0938	1	73	1	0	0	C/C
Control-0939	2	66	0	0	1	C/C
Control-0940	1	79	0	0	0	C/C
Control-0941	1	72	0	0	1	C/C
Control-0942	2	61	0	0	0	G/C
Control-0943	1	80	0	1	1	G/C
Control-0944	1	66	1	0	0	G/C
Control-0945	1	74	0	1	0	G/C
Control-0946	2	61	0	0	0	C/C
Control-0947	1	70	0	0	1	C/C
Control-0948	1	73	0	0	0	G/C
Control-0949	1	75	0	1	0	G/C
Control-0950	1	71	0	0	1	C/C
Control-0951	1	69	1	1	1	C/C
Control-0952	1	79	0	0	0	C/C
Control-0953	1	81	1	0	0	G/C
Control-0954	1	60	0	0	0	G/G
Control-0955	1	68	0	0	1	G/C
Control-0956	1	77	0	0	1	C/C
Control-0957	1	56	0	0	1	G/C
Control-0958	1	66	0	0	0	C/C
Control-0959	2	51	0	0	0	C/C
Control-0960	1	70	1	1	1	C/C
Control-0961	1	70	0	0	0	G/G
Control-0962	1	47	1	1	1	G/C
Control-0963	1	83	0	0	0	G/C
Control-0964	2	72	0	0	0	G/C
Control-0965	1	82	0	0	1	C/C
Control-0966	1	80	0	0	0	G/C
Control-0967	2	74	0	0	0	G/C
Control-0968	2	59	0	0	0	G/G
Control-0969	1	69	0	0	1	G/C

Control-0970	1	81	0	0	1	C/C
Control-0971	1	74	0	0	0	G/C
Control-0972	1	64	0	0	0	C/C
Control-0973	1	77	0	0	0	G/C
Control-0974	2	66	0	0	0	G/C
Control-0975	1	71	0	1	1	G/G
Control-0976	2	72	0	0	1	C/C
Control-0977	1	65	0	1	1	C/C
Control-0978	2	67	0	0	1	G/C
Control-0979	2	79	0	0	1	G/C
Control-0980	1	79	0	1	0	C/C
Control-0981	2	59	0	0	0	G/C
Control-0982	2	53	0	0	0	C/C
Control-0983	1	66	0	1	1	C/C
Control-0984	2	75	0	0	1	G/C
Control-0985	1	50	0	0	1	C/C
Control-0986	1	67	0	0	1	C/C
Control-0987	1	83	0	0	1	G/G
Control-0988	2	50	0	0	0	G/C
Control-0989	1	69	0	1	1	G/G
Control-0990	2	69	0	0	0	G/C
Control-0991	1	63	1	0	0	C/C
Control-0992	1	52	0	0	0	G/G
Control-0993	2	69	0	0	0	C/C
Control-0994	1	75	0	0	0	G/C
Control-0995	1	76	0	0	0	C/C
Control-0996	1	71	0	0	1	C/C
Control-0997	1	78	0	0	1	C/C
Control-0998	1	61	0	1	0	C/C
Control-0999	1	73	0	0	1	G/C
Control-1000	2	68	0	0	0	G/C
Control-1001	1	61	1	1	1	G/C
Control-1002	2	61	0	0	0	G/C
Control-1003	1	73	0	0	1	G/C
Control-1004	1	62	0	0	1	C/C
Control-1005	1	52	1	0	0	C/C
Control-1006	1	62	1	1	1	C/C
Control-1007	2	53	0	0	1	G/C
Control-1008	1	74	0	0	0	C/C
Control-1009	1	58	1	1	0	G/G
Control-1010	1	77	0	1	0	C/C
Control-1011	2	74	0	0	1	G/G
Control-1012	2	67	0	0	1	G/C
Control-1013	1	77	0	0	0	C/C
Control-1014	1	79	0	0	1	G/C
Control-1015	1	76	0	0	1	G/C
Control-1016	1	44	1	0	0	C/C
Control-1017	1	49	0	0	0	C/C
Control-1018	2	64	0	0	0	G/G
Control-1019	2	61	0	0	1	G/C
Control-1020	2	68	0	0	1	C/C
Control-1021	2	62	0	0	1	G/G
Control-1022	1	50	0	1	0	G/C
Control-1023	1	46	0	0	1	C/C
Control-1024	1	51	1	1	1	G/C
Control-1025	1	59	0	0	0	G/C
Control-1026	1	70	0	0	1	G/G
Control-1027	1	69	0	0	1	G/C
Control-1028	1	68	1	0	1	G/G
Control-1029	1	58	0	1	1	G/C
Control-1030	1	72	1	0	1	G/G
Control-1031	1	81	1	0	1	G/C
Control-1032	1	66	1	0	1	C/C
Control-1033	2	48	0	0	0	C/C
Control-1034	1	76	0	1	0	G/C
Control-1035	1	61	0	0	1	G/G

Control-1036	1	77	0	1	0	G/C
Control-1037	1	67	0	0	1	G/C
Control-1038	1	75	0	0	1	G/C
Control-1039	1	61	0	1	1	G/G
Control-1040	1	74	0	1	1	G/C
Control-1041	1	74	0	0	0	C/C
Control-1042	1	61	1	0	1	G/C
Control-1043	1	62	0	1	0	G/G
Control-1044	1	72	0	1	1	C/C
Control-1045	2	63	0	0	1	C/C
Control-1046	1	60	1	0	1	C/C
Control-1047	2	41	0	0	0	C/C
Control-1048	1	58	1	0	1	G/G
Control-1049	1	61	1	1	0	C/C
Control-1050	2	49	0	0	0	C/C
Control-1051	2	66	0	0	0	G/G
Control-1052	1	68	0	0	0	G/G
Control-1053	1	62	1	0	0	C/C
Control-1054	1	77	0	0	0	G/C
Control-1055	2	45	0	0	0	C/C
Control-1056	1	71	0	1	0	G/C
Control-1057	1	59	0	0	1	C/C
Control-1058	1	77	1	1	1	G/G
Control-1059	2	45	0	0	1	G/C
Control-1060	1	71	0	0	1	G/C
Control-1061	1	61	0	0	0	G/G
Control-1062	1	66	1	1	0	C/C
Control-1063	2	46	0	0	1	G/C
Control-1064	2	67	0	0	0	C/C
Control-1065	1	81	1	0	0	C/C
Control-1066	1	52	0	0	1	G/C
Control-1067	1	65	1	0	0	G/C
Control-1068	1	53	0	0	0	G/C
Control-1069	2	65	0	0	0	G/C
Control-1070	1	64	0	1	1	C/C
Control-1071	1	61	1	1	1	G/C
Control-1072	1	71	1	0	0	C/C
Control-1073	1	75	0	0	1	C/C
Control-1074	2	74	0	0	0	C/C
Control-1075	1	58	1	1	1	G/C
Control-1076	2	62	0	0	0	G/C
Control-1077	1	79	0	0	0	G/C
Control-1078	1	81	0	0	0	G/C
Control-1079	1	57	1	0	1	C/C
Control-1080	1	75	0	0	0	G/C
Control-1081	1	64	1	0	0	G/C
Control-1082	2	55	0	0	0	G/C
Control-1083	2	59	0	0	1	C/C
Control-1084	2	59	1	1	1	C/C
Control-1085	2	57	0	0	1	C/C
Control-1086	1	59	1	0	0	G/G
Control-1087	2	64	0	0	0	C/C
Control-1088	1	59	0	0	1	G/G
Control-1089	2	58	0	0	1	C/C
Control-1090	2	72	0	0	1	G/C
Control-1091	1	60	1	1	0	G/C
Control-1092	2	64	0	0	1	C/C
Control-1093	1	78	0	1	1	G/G
Control-1094	1	60	0	0	0	G/C
Control-1095	1	75	0	0	0	G/C
Control-1096	1	53	0	0	0	C/C
Control-1097	1	82	1	0	0	G/C
Control-1098	1	63	0	0	1	G/C
Control-1099	1	46	0	0	1	C/C
Control-1100	1	44	0	0	1	G/C
Control-1101	1	66	1	0	0	G/C

Control-1102	1	74	0	0	0	C/C
Control-1103	1	53	1	1	0	G/C
Control-1104	2	74	0	0	0	G/C
Control-1105	2	62	0	0	0	G/C
Control-1106	2	63	0	0	0	C/C
Control-1107	1	74	0	0	1	G/G
Control-1108	1	51	1	1	1	G/C
Control-1109	1	58	0	1	0	G/C
Control-1110	2	75	0	0	1	G/C
Control-1111	1	57	0	0	1	G/C
Control-1112	2	56	0	0	1	G/C
Control-1113	1	67	0	0	1	G/C
Control-1114	1	67	1	1	1	C/C
Control-1115	1	57	0	0	0	G/C
Control-1116	1	54	0	0	1	C/C
Control-1117	1	52	0	0	0	G/G
Control-1118	2	52	0	0	0	G/G
Control-1119	1	53	0	0	0	G/G
Control-1120	1	59	0	0	0	G/C
Control-1121	1	61	0	0	0	G/C
Control-1122	2	66	0	0	0	C/C
Control-1123	1	63	0	0	0	G/C
Control-1124	1	57	0	0	1	G/C
Control-1125	1	56	0	0	1	G/C
Control-1126	1	55	0	0	0	G/C
Control-1127	2	53	0	0	0	G/C
Control-1128	2	65	0	0	0	G/C
Control-1129	2	72	0	0	0	G/C
Control-1130	2	68	0	0	1	C/C
Control-1131	1	62	0	0	1	G/G
Control-1132	1	52	0	0	1	C/C
Control-1133	1	59	0	0	1	C/C
Control-1134	1	56	0	0	1	G/C
Control-1135	1	58	0	0	0	G/C
Control-1136	2	49	0	0	1	C/C
Control-1137	1	52	0	0	1	C/C
Control-1138	2	56	0	0	0	G/C
Control-1139	2	64	0	0	1	C/C
Control-1140	2	68	0	0	0	G/G
Control-1141	1	48	0	0	0	G/C
Control-1142	1	64	1	1	0	G/C
Control-1143	2	53	0	0	0	C/C
Control-1144	1	49	0	0	0	C/C
Control-1145	1	61	1	0	0	C/C
Control-1146	2	54	0	0	0	G/C
Control-1147	1	57	1	0	0	C/C
Control-1148	1	58	1	0	1	G/G
Control-1149	2	49	0	0	1	C/C
Control-1150	2	61	0	0	0	G/C
Control-1151	2	59	0	0	0	G/C
Control-1152	2	67	0	0	0	G/C
Control-1153	2	68	0	0	1	G/C
Control-1154	2	57	0	0	0	C/C
Control-1155	2	71	0	0	1	G/G
Control-1156	1	55	0	0	1	G/C
Control-1157	1	61	1	1	0	C/C
Control-1158	1	53	0	0	1	G/C
Control-1159	1	59	1	0	1	G/C
Control-1160	2	61	0	0	0	G/C
Control-1161	2	70	0	0	1	C/C
Control-1162	2	52	0	0	1	G/C
Control-1163	2	58	0	0	0	G/C
Control-1164	2	64	0	0	0	C/C
Control-1165	2	59	0	0	1	G/G
Control-1166	2	75	0	0	1	G/C
Control-1167	2	58	0	0	0	C/C

Control-1168	2	62	0	0	0	G/G
Control-1169	2	63	0	0	1	G/C
Control-1170	1	61	0	1	0	G/G
Control-1171	2	59	0	0	0	G/C
Control-1172	2	77	0	0	1	C/C
Control-1173	2	59	0	0	0	C/C
Control-1174	2	59	0	0	0	G/C
Control-1175	1	61	1	0	0	C/C
Control-1176	1	60	0	1	0	G/C
Control-1177	2	63	0	0	1	G/C
Control-1178	2	65	0	0	1	C/C
Control-1179	2	80	0	0	1	C/C
Control-1180	1	59	1	0	1	C/C
Control-1181	1	64	0	0	1	C/C
Control-1182	1	63	1	1	1	C/C
Control-1183	2	64	0	0	0	C/C
Control-1184	1	57	0	0	1	G/C
Control-1185	2	67	0	0	0	G/C
Control-1186	2	72	0	0	0	G/C
Control-1187	2	63	0	0	1	G/C
Control-1188	2	66	0	0	0	G/G
Control-1189	2	63	0	0	0	G/C
Control-1190	2	60	0	0	1	G/C
Control-1191	2	59	0	0	1	G/C
Control-1192	2	63	0	0	1	G/C
Control-1193	2	59	0	0	1	C/C
Control-1194	1	62	0	0	0	G/G
Control-1195	2	62	0	0	0	C/C
Control-1196	1	63	1	1	0	G/C
Control-1197	2	56	0	0	0	C/C
Control-1198	1	60	0	0	0	G/C
Control-1199	1	54	0	0	0	G/C
Control-1200	1	58	0	0	1	G/G
Control-1201	1	51	0	0	1	C/C
Control-1202	1	55	0	0	0	G/C
Control-1203	1	59	0	0	1	G/C
Control-1204	1	54	0	0	1	?
Control-1205	2	55	0	0	1	C/C
Control-1206	1	59	0	0	0	C/C
Control-1207	1	54	0	0	0	G/C
Control-1208	1	69	1	1	0	G/C
Control-1209	2	62	0	0	0	G/C
Control-1210	2	62	0	0	1	C/C
Control-1211	1	54	0	0	0	G/C
Control-1212	1	57	0	0	0	G/C
Control-1213	1	52	0	0	0	?
Control-1214	1	54	0	0	1	C/C
Control-1215	1	56	0	0	1	C/C
Control-1216	1	52	0	0	0	G/C
Control-1217	1	50	0	0	0	C/C
Control-1218	1	62	0	0	0	G/C
Control-1219	1	40	0	0	1	C/C
Control-1220	1	49	0	0	1	G/C
Control-1221	1	53	0	0	1	G/C
Control-1222	1	45	0	0	0	G/G
Control-1223	1	48	0	0	0	G/G
Control-1224	1	43	0	0	0	C/C
Control-1225	1	61	0	0	1	G/G
Control-1226	1	51	0	0	1	G/C
Control-1227	2	72	0	0	1	G/C
Control-1228	1	55	0	0	1	G/G
Control-1229	2	50	0	0	1	G/C
Control-1230	2	56	0	0	1	G/G
Control-1231	1	53	1	1	0	C/C
Control-1232	2	55	0	0	0	G/C
Control-1233	1	80	1	0	1	C/C

Control-1234	1	62	0	0	1	G/C
Control-1235	2	64	0	0	1	C/C
Control-1236	1	49	0	0	1	G/G
Control-1237	2	61	0	0	1	G/C
Control-1238	1	51	0	0	1	G/C
Control-1239	1	47	0	0	0	G/G
Control-1240	1	49	0	0	0	G/C
Control-1241	2	62	0	0	1	G/G
Control-1242	2	68	0	0	1	C/C
Control-1243	1	40	0	0	0	C/C
Control-1244	1	59	0	0	0	G/C
Control-1245	1	49	0	0	0	C/C
Control-1246	1	63	0	0	0	C/C
Control-1247	1	49	0	0	1	C/C
Control-1248	2	67	0	0	1	C/C
Control-1249	1	42	1	1	0	G/G
Control-1250	1	41	0	0	0	G/G
Control-1251	2	56	0	0	1	G/G
Control-1252	1	60	1	1	0	G/C
Control-1253	1	65	1	0	0	C/C
Control-1254	2	64	0	0	1	C/C
Control-1255	1	55	0	0	0	C/C
Control-1256	2	40	0	0	0	C/C
Control-1257	1	50	0	1	0	C/C
Control-1258	2	79	0	0	1	G/C
Control-1259	1	50	1	0	0	G/C
Control-1260	2	65	0	0	0	G/C
Control-1261	2	53	0	0	1	G/C
Control-1262	2	62	0	0	0	C/C
Control-1263	2	64	0	0	0	C/C
Control-1264	2	55	0	0	0	G/C
Control-1265	2	63	0	0	1	G/G
Control-1266	2	57	0	0	1	C/C
Control-1267	2	56	0	0	0	C/C
Control-1268	2	62	0	0	0	G/C
Control-1269	2	64	0	0	0	G/G
Control-1270	2	60	0	0	1	C/C
Control-1271	2	51	0	0	1	G/G
Control-1272	2	61	0	0	1	C/C
Control-1273	2	52	0	0	0	G/C
Control-1274	2	64	0	0	0	G/C
Control-1275	2	56	0	0	0	G/C
Control-1276	2	60	0	0	0	G/C
Control-1277	2	65	0	0	0	G/C
Control-1278	2	62	0	0	0	G/C
Control-1279	2	58	0	0	0	C/C
Control-1280	2	65	0	0	1	G/C
Control-1281	2	59	0	0	0	C/C
Control-1282	2	62	0	0	0	?
Control-1283	2	60	0	0	1	G/C
Control-1284	2	53	0	0	1	C/C
Control-1285	2	63	0	0	1	C/C
Control-1286	2	64	0	0	0	C/C
Control-1287	2	56	0	0	0	G/C
Control-1288	2	61	0	0	1	G/C
Control-1289	2	59	0	0	0	G/C
Control-1290	2	57	0	0	0	C/C
Control-1291	2	64	0	0	1	C/C
Control-1292	2	60	0	0	0	C/C
Control-1293	2	65	0	0	0	C/C
Control-1294	2	32	0	0	0	C/C
Control-1295	2	50	0	0	1	C/C
Control-1296	2	64	0	0	1	G/G
Control-1297	2	56	0	0	0	G/G
Control-1298	2	60	0	0	1	G/C
Control-1299	2	58	0	0	1	C/C

Control-1300	2	63	0	0	1	G/C
Control-1301	2	64	0	0	1	G/C
Control-1302	2	50	0	0	1	C/C
Control-1303	2	52	0	0	1	G/C
